# Supplementary material for: The impact of missing data rates and imputation methods on the assumption of unidimensionality
Source: PLoS One. 2025 Apr 30;20(4):e0321344. doi: 10.1371/journal.pone.0321344 (PMC12043241; doi:10.1371/journal.pone.0321344)
Supplement: Appendix 1 — (DOCX) [file pone.0321344.s001.docx]

Appendix (1)

Corrected Item-Total Correlation (CITC) DUE TO PERCENTAGE OF MISSINGNESS

| **Item** | **METHOD** | **0%** | **2%** | **4%** | **6%** | **7%** | **8%** | **9%** | **10%** | **11%** | **12%** | **13%** | **14%** | **15%** | **20%** | **25%** | **30%** | **35%** | **40%** | **45%** | **50%** | **MEAN** |
| --- | --- | --- | --- | --- | --- | --- | --- | --- | --- | --- | --- | --- | --- | --- | --- | --- | --- | --- | --- | --- | --- | --- |
| 1 | CIM | 0.76 | 0.76 | 0.76 | 0.77 | 0.77 | 0.78 | 0.78 | 0.78 | 0.78 | 0.79 | 0.79 | 0.79 | 0.79 | 0.81 | 0.82 | 0.83 | 0.84 | 0.86 | 0.87 | 0.88 |  |
|  | EM | 0.76 | 0.76 | 0.76 | 0.76 | 0.76 | 0.76 | 0.76 | 0.76 | 0.77 | 0.77 | 0.77 | 0.77 | 0.77 | 0.77 | 0.78 | 0.78 | 0.79 | 0.80 | 0.80 | 0.80 | 0.7729 |
|  | MI | 0.76 | 0.76 | 0.76 | 0.76 | 0.76 | 0.76 | 0.78 | 0.76 | 0.77 | 0.77 | 0.77 | 0.77 | 0.77 | 0.77 | 0.78 | 0.78 | 0.79 | 0.80 | 0.79 | 0.80 | 0.7736 |
| 2 | CIM | 0.86 | 0.87 | 0.87 | 0.87 | 0.87 | 0.88 | 0.88 | 0.88 | 0.88 | 0.88 | 0.88 | 0.88 | 0.89 | 0.89 | 0.90 | 0.91 | 0.92 | 0.92 | 0.93 | 0.93 | 0.8895 |
|  | EM | 0.86 | 0.86 | 0.87 | 0.87 | 0.87 | 0.87 | 0.87 | 0.87 | 0.87 | 0.87 | 0.87 | 0.87 | 0.87 | 0.88 | 0.87 | 0.88 | 0.88 | 0.89 | 0.89 | 0.89 | 0.8732 |
|  | MI | 0.86 | 0.86 | 0.87 | 0.87 | 0.87 | 0.87 | 0.88 | 0.87 | 0.87 | 0.87 | 0.87 | 0.87 | 0.87 | 0.88 | 0.87 | 0.88 | 0.88 | 0.89 | 0.89 | 0.89 | 0.8737 |
| 3 | CIM | 0.88 | 0.88 | 0.88 | 0.88 | 0.89 | 0.89 | 0.89 | 0.89 | 0.89 | 0.89 | 0.89 | 0.89 | 0.89 | 0.90 | 0.90 | 0.91 | 0.91 | 0.92 | 0.93 | 0.93 | 0.8980 |
|  | EM | 0.88 | 0.88 | 0.88 | 0.88 | 0.89 | 0.89 | 0.89 | 0.89 | 0.89 | 0.89 | 0.89 | 0.89 | 0.89 | 0.90 | 0.90 | 0.90 | 0.91 | 0.92 | 0.92 | 0.92 | 0.8954 |
|  | MI | 0.88 | 0.88 | 0.88 | 0.88 | 0.89 | 0.89 | 0.89 | 0.89 | 0.89 | 0.89 | 0.89 | 0.89 | 0.89 | 0.90 | 0.90 | 0.90 | 0.91 | 0.91 | 0.92 | 0.92 | 0.8954 |
| 4 | CIM | 0.89 | 0.89 | 0.89 | 0.89 | 0.89 | 0.89 | 0.90 | 0.90 | 0.90 | 0.90 | 0.90 | 0.90 | 0.90 | 0.90 | 0.90 | 0.91 | 0.91 | 0.92 | 0.92 | 0.92 | 0.9014 |
|  | EM | 0.89 | 0.89 | 0.89 | 0.90 | 0.90 | 0.90 | 0.90 | 0.90 | 0.90 | 0.90 | 0.90 | 0.90 | 0.90 | 0.91 | 0.91 | 0.91 | 0.92 | 0.93 | 0.93 | 0.93 | 0.9057 |
|  | MI | 0.89 | 0.89 | 0.89 | 0.90 | 0.90 | 0.90 | 0.90 | 0.90 | 0.90 | 0.90 | 0.90 | 0.90 | 0.90 | 0.91 | 0.91 | 0.91 | 0.92 | 0.93 | 0.93 | 0.93 | 0.9055 |
| 5 | CIM | 0.84 | 0.84 | 0.85 | 0.85 | 0.85 | 0.85 | 0.86 | 0.86 | 0.86 | 0.86 | 0.86 | 0.86 | 0.86 | 0.87 | 0.88 | 0.88 | 0.90 | 0.91 | 0.91 | 0.92 | 0.8690 |
|  | EM | 0.84 | 0.84 | 0.85 | 0.84 | 0.85 | 0.85 | 0.85 | 0.85 | 0.85 | 0.85 | 0.85 | 0.85 | 0.85 | 0.86 | 0.86 | 0.87 | 0.87 | 0.88 | 0.89 | 0.89 | 0.8576 |
|  | MI | 0.84 | 0.84 | 0.85 | 0.84 | 0.85 | 0.85 | 0.86 | 0.85 | 0.85 | 0.85 | 0.85 | 0.85 | 0.85 | 0.86 | 0.86 | 0.87 | 0.87 | 0.88 | 0.89 | 0.89 | 0.8576 |
| 6 | CIM | 0.89 | 0.89 | 0.89 | 0.90 | 0.90 | 0.90 | 0.90 | 0.90 | 0.90 | 0.91 | 0.90 | 0.91 | 0.91 | 0.91 | 0.92 | 0.93 | 0.93 | 0.94 | 0.94 | 0.95 | 0.9111 |
|  | EM | 0.89 | 0.89 | 0.89 | 0.89 | 0.89 | 0.89 | 0.89 | 0.89 | 0.89 | 0.90 | 0.89 | 0.89 | 0.90 | 0.90 | 0.90 | 0.90 | 0.90 | 0.91 | 0.91 | 0.90 | 0.8963 |
|  | MI | 0.89 | 0.89 | 0.89 | 0.89 | 0.89 | 0.89 | 0.90 | 0.89 | 0.89 | 0.90 | 0.89 | 0.89 | 0.90 | 0.90 | 0.90 | 0.90 | 0.90 | 0.91 | 0.91 | 0.91 | 0.8970 |
| 7 | CIM | 0.84 | 0.84 | 0.84 | 0.84 | 0.84 | 0.85 | 0.85 | 0.85 | 0.85 | 0.85 | 0.85 | 0.85 | 0.86 | 0.86 | 0.87 | 0.88 | 0.88 | 0.90 | 0.90 | 0.91 | 0.8611 |
|  | EM | 0.84 | 0.84 | 0.84 | 0.84 | 0.84 | 0.84 | 0.85 | 0.85 | 0.85 | 0.85 | 0.85 | 0.85 | 0.85 | 0.86 | 0.86 | 0.87 | 0.87 | 0.88 | 0.89 | 0.89 | 0.8555 |
|  | MI | 0.84 | 0.84 | 0.84 | 0.84 | 0.84 | 0.84 | 0.85 | 0.85 | 0.85 | 0.85 | 0.85 | 0.85 | 0.85 | 0.85 | 0.86 | 0.87 | 0.87 | 0.88 | 0.89 | 0.89 | 0.8554 |
| 8 | CIM | 0.66 | 0.67 | 0.67 | 0.68 | 0.69 | 0.69 | 0.69 | 0.70 | 0.69 | 0.70 | 0.71 | 0.71 | 0.71 | 0.72 | 0.74 | 0.75 | 0.77 | 0.79 | 0.81 | 0.83 | 0.7224 |
|  | EM | 0.66 | 0.67 | 0.67 | 0.68 | 0.68 | 0.68 | 0.68 | 0.69 | 0.68 | 0.69 | 0.69 | 0.69 | 0.69 | 0.70 | 0.72 | 0.72 | 0.72 | 0.74 | 0.76 | 0.77 | 0.6987 |
|  | MI | 0.66 | 0.67 | 0.67 | 0.68 | 0.67 | 0.68 | 0.69 | 0.69 | 0.68 | 0.69 | 0.69 | 0.69 | 0.69 | 0.70 | 0.72 | 0.72 | 0.72 | 0.74 | 0.76 | 0.77 | 0.6994 |
| 9 | CIM | 0.83 | 0.83 | 0.83 | 0.83 | 0.83 | 0.83 | 0.84 | 0.83 | 0.84 | 0.84 | 0.83 | 0.84 | 0.84 | 0.84 | 0.84 | 0.85 | 0.85 | 0.86 | 0.87 | 0.87 | 0.8417 |
|  | EM | 0.83 | 0.83 | 0.83 | 0.83 | 0.83 | 0.83 | 0.83 | 0.83 | 0.83 | 0.83 | 0.83 | 0.83 | 0.84 | 0.84 | 0.84 | 0.84 | 0.85 | 0.85 | 0.85 | 0.85 | 0.8369 |
|  | MI | 0.83 | 0.83 | 0.83 | 0.83 | 0.83 | 0.83 | 0.84 | 0.83 | 0.83 | 0.83 | 0.84 | 0.83 | 0.84 | 0.84 | 0.84 | 0.84 | 0.85 | 0.85 | 0.85 | 0.85 | 0.8371 |
| 10 | CIM | 0.81 | 0.81 | 0.81 | 0.82 | 0.82 | 0.82 | 0.82 | 0.82 | 0.83 | 0.83 | 0.83 | 0.83 | 0.83 | 0.84 | 0.84 | 0.85 | 0.86 | 0.87 | 0.89 | 0.89 | 0.8374 |
|  | EM | 0.81 | 0.81 | 0.81 | 0.82 | 0.82 | 0.82 | 0.82 | 0.82 | 0.82 | 0.82 | 0.83 | 0.82 | 0.83 | 0.84 | 0.84 | 0.85 | 0.85 | 0.86 | 0.87 | 0.88 | 0.8323 |
|  | MI | 0.81 | 0.81 | 0.81 | 0.82 | 0.82 | 0.82 | 0.82 | 0.82 | 0.82 | 0.82 | 0.83 | 0.82 | 0.83 | 0.84 | 0.84 | 0.85 | 0.85 | 0.86 | 0.87 | 0.88 | 0.8321 |
| 11 | CIM | 0.81 | 0.81 | 0.81 | 0.82 | 0.82 | 0.82 | 0.82 | 0.82 | 0.82 | 0.83 | 0.82 | 0.83 | 0.83 | 0.84 | 0.84 | 0.85 | 0.85 | 0.87 | 0.87 | 0.88 | 0.8351 |
|  | EM | 0.81 | 0.81 | 0.82 | 0.82 | 0.82 | 0.82 | 0.82 | 0.82 | 0.82 | 0.83 | 0.82 | 0.83 | 0.83 | 0.84 | 0.84 | 0.85 | 0.85 | 0.86 | 0.87 | 0.87 | 0.8324 |
|  | MI | 0.81 | 0.81 | 0.82 | 0.82 | 0.82 | 0.82 | 0.82 | 0.82 | 0.82 | 0.83 | 0.82 | 0.83 | 0.83 | 0.83 | 0.84 | 0.84 | 0.85 | 0.86 | 0.86 | 0.87 | 0.8319 |
| 12 | CIM | 0.89 | 0.89 | 0.89 | 0.89 | 0.89 | 0.89 | 0.89 | 0.89 | 0.89 | 0.89 | 0.89 | 0.89 | 0.89 | 0.90 | 0.90 | 0.90 | 0.90 | 0.90 | 0.90 | 0.91 | 0.8954 |
|  | EM | 0.89 | 0.89 | 0.89 | 0.89 | 0.90 | 0.90 | 0.90 | 0.90 | 0.90 | 0.90 | 0.90 | 0.90 | 0.90 | 0.90 | 0.90 | 0.91 | 0.91 | 0.91 | 0.91 | 0.92 | 0.9005 |
|  | MI | 0.89 | 0.89 | 0.89 | 0.89 | 0.90 | 0.90 | 0.89 | 0.90 | 0.90 | 0.90 | 0.90 | 0.90 | 0.90 | 0.90 | 0.90 | 0.91 | 0.91 | 0.91 | 0.91 | 0.92 | 0.9003 |
| 13 | CIM | 0.86 | 0.86 | 0.87 | 0.87 | 0.87 | 0.87 | 0.87 | 0.87 | 0.87 | 0.87 | 0.88 | 0.88 | 0.88 | 0.89 | 0.89 | 0.89 | 0.90 | 0.91 | 0.91 | 0.92 | 0.8821 |
|  | EM | 0.86 | 0.87 | 0.87 | 0.87 | 0.87 | 0.87 | 0.87 | 0.87 | 0.87 | 0.87 | 0.88 | 0.87 | 0.87 | 0.88 | 0.88 | 0.89 | 0.89 | 0.90 | 0.89 | 0.90 | 0.8771 |
|  | MI | 0.86 | 0.87 | 0.87 | 0.87 | 0.87 | 0.87 | 0.87 | 0.87 | 0.87 | 0.87 | 0.88 | 0.87 | 0.87 | 0.88 | 0.88 | 0.89 | 0.89 | 0.89 | 0.89 | 0.90 | 0.8771 |
| 14 | CIM | 0.92 | 0.92 | 0.92 | 0.93 | 0.93 | 0.93 | 0.93 | 0.93 | 0.93 | 0.93 | 0.93 | 0.93 | 0.93 | 0.93 | 0.93 | 0.93 | 0.93 | 0.94 | 0.94 | 0.95 | 0.9291 |
|  | EM | 0.92 | 0.93 | 0.93 | 0.93 | 0.93 | 0.93 | 0.93 | 0.93 | 0.93 | 0.93 | 0.93 | 0.93 | 0.93 | 0.93 | 0.94 | 0.94 | 0.94 | 0.94 | 0.94 | 0.95 | 0.9317 |
|  | MI | 0.92 | 0.93 | 0.93 | 0.93 | 0.93 | 0.93 | 0.93 | 0.93 | 0.93 | 0.93 | 0.93 | 0.93 | 0.93 | 0.93 | 0.93 | 0.94 | 0.94 | 0.94 | 0.94 | 0.95 | 0.9315 |
| 15 | CIM | 0.87 | 0.87 | 0.87 | 0.88 | 0.88 | 0.88 | 0.88 | 0.88 | 0.88 | 0.89 | 0.89 | 0.89 | 0.89 | 0.90 | 0.90 | 0.91 | 0.92 | 0.92 | 0.93 | 0.94 | 0.8945 |
|  | EM | 0.87 | 0.87 | 0.87 | 0.87 | 0.87 | 0.88 | 0.88 | 0.88 | 0.88 | 0.88 | 0.88 | 0.88 | 0.88 | 0.88 | 0.89 | 0.89 | 0.89 | 0.89 | 0.89 | 0.90 | 0.8802 |
|  | MI | 0.87 | 0.87 | 0.87 | 0.87 | 0.87 | 0.88 | 0.88 | 0.88 | 0.88 | 0.88 | 0.88 | 0.88 | 0.88 | 0.88 | 0.89 | 0.89 | 0.89 | 0.89 | 0.89 | 0.90 | 0.8804 |
| 16 | CIM | 0.82 | 0.82 | 0.82 | 0.83 | 0.83 | 0.83 | 0.83 | 0.84 | 0.84 | 0.84 | 0.84 | 0.84 | 0.84 | 0.85 | 0.86 | 0.87 | 0.88 | 0.89 | 0.90 | 0.91 | 0.8507 |
|  | EM | 0.82 | 0.82 | 0.82 | 0.82 | 0.82 | 0.82 | 0.83 | 0.83 | 0.83 | 0.83 | 0.83 | 0.83 | 0.83 | 0.83 | 0.84 | 0.84 | 0.85 | 0.86 | 0.86 | 0.87 | 0.8351 |
|  | MI | 0.82 | 0.82 | 0.82 | 0.82 | 0.82 | 0.82 | 0.83 | 0.83 | 0.83 | 0.83 | 0.83 | 0.83 | 0.83 | 0.83 | 0.84 | 0.84 | 0.85 | 0.86 | 0.86 | 0.87 | 0.8351 |
| 17 | CIM | 0.94 | 0.94 | 0.94 | 0.95 | 0.95 | 0.95 | 0.95 | 0.95 | 0.95 | 0.95 | 0.95 | 0.95 | 0.95 | 0.95 | 0.95 | 0.95 | 0.95 | 0.96 | 0.96 | 0.96 | 0.9494 |
|  | EM | 0.94 | 0.95 | 0.95 | 0.95 | 0.95 | 0.95 | 0.95 | 0.95 | 0.95 | 0.95 | 0.95 | 0.95 | 0.95 | 0.95 | 0.95 | 0.95 | 0.95 | 0.95 | 0.96 | 0.96 | 0.9488 |
|  | MI | 0.94 | 0.95 | 0.95 | 0.95 | 0.95 | 0.95 | 0.95 | 0.95 | 0.95 | 0.95 | 0.95 | 0.95 | 0.95 | 0.95 | 0.95 | 0.95 | 0.95 | 0.95 | 0.96 | 0.96 | 0.9487 |
| 18 | CIM | 0.89 | 0.89 | 0.89 | 0.89 | 0.89 | 0.89 | 0.89 | 0.89 | 0.89 | 0.89 | 0.89 | 0.89 | 0.89 | 0.89 | 0.90 | 0.90 | 0.91 | 0.91 | 0.92 | 0.92 | 0.8958 |
|  | EM | 0.89 | 0.89 | 0.89 | 0.89 | 0.89 | 0.89 | 0.89 | 0.89 | 0.89 | 0.89 | 0.89 | 0.89 | 0.89 | 0.89 | 0.90 | 0.90 | 0.90 | 0.91 | 0.91 | 0.91 | 0.8945 |
|  | MI | 0.89 | 0.89 | 0.89 | 0.89 | 0.89 | 0.89 | 0.89 | 0.89 | 0.89 | 0.89 | 0.89 | 0.89 | 0.89 | 0.89 | 0.90 | 0.90 | 0.90 | 0.91 | 0.91 | 0.91 | 0.8944 |
| 19 | CIM | 0.75 | 0.76 | 0.76 | 0.77 | 0.77 | 0.77 | 0.78 | 0.78 | 0.78 | 0.79 | 0.79 | 0.79 | 0.80 | 0.80 | 0.82 | 0.83 | 0.85 | 0.86 | 0.88 | 0.89 | 0.8027 |
|  | EM | 0.75 | 0.75 | 0.76 | 0.76 | 0.76 | 0.76 | 0.76 | 0.77 | 0.76 | 0.77 | 0.77 | 0.77 | 0.77 | 0.77 | 0.78 | 0.79 | 0.80 | 0.81 | 0.82 | 0.82 | 0.7750 |
|  | MI | 0.75 | 0.75 | 0.76 | 0.76 | 0.76 | 0.76 | 0.78 | 0.77 | 0.76 | 0.77 | 0.77 | 0.77 | 0.77 | 0.77 | 0.78 | 0.79 | 0.80 | 0.81 | 0.81 | 0.82 | 0.7756 |
| 20 | CIM | 0.84 | 0.84 | 0.84 | 0.85 | 0.85 | 0.85 | 0.85 | 0.85 | 0.86 | 0.86 | 0.86 | 0.86 | 0.86 | 0.87 | 0.88 | 0.89 | 0.89 | 0.90 | 0.91 | 0.92 | 0.8669 |
|  | EM | 0.84 | 0.84 | 0.84 | 0.85 | 0.85 | 0.85 | 0.85 | 0.85 | 0.85 | 0.85 | 0.85 | 0.85 | 0.85 | 0.86 | 0.86 | 0.86 | 0.87 | 0.87 | 0.88 | 0.88 | 0.8538 |
|  | MI | 0.84 | 0.84 | 0.84 | 0.85 | 0.85 | 0.85 | 0.85 | 0.85 | 0.85 | 0.85 | 0.85 | 0.85 | 0.85 | 0.86 | 0.86 | 0.86 | 0.87 | 0.87 | 0.87 | 0.88 | 0.8539 |
| 21 | CIM | 0.92 | 0.92 | 0.92 | 0.93 | 0.93 | 0.93 | 0.93 | 0.93 | 0.93 | 0.93 | 0.93 | 0.93 | 0.93 | 0.94 | 0.94 | 0.94 | 0.95 | 0.96 | 0.96 | 0.96 | 0.9363 |
|  | EM | 0.92 | 0.92 | 0.92 | 0.93 | 0.93 | 0.93 | 0.93 | 0.93 | 0.93 | 0.93 | 0.93 | 0.93 | 0.93 | 0.93 | 0.93 | 0.93 | 0.93 | 0.94 | 0.94 | 0.94 | 0.9285 |
|  | MI | 0.92 | 0.92 | 0.92 | 0.93 | 0.93 | 0.93 | 0.93 | 0.93 | 0.93 | 0.93 | 0.93 | 0.93 | 0.93 | 0.93 | 0.93 | 0.93 | 0.93 | 0.94 | 0.94 | 0.94 | 0.9288 |
| 22 | CIM | 0.89 | 0.89 | 0.90 | 0.90 | 0.90 | 0.90 | 0.90 | 0.90 | 0.90 | 0.90 | 0.90 | 0.90 | 0.91 | 0.91 | 0.91 | 0.92 | 0.92 | 0.92 | 0.93 | 0.94 | 0.9077 |
|  | EM | 0.89 | 0.90 | 0.90 | 0.90 | 0.90 | 0.90 | 0.90 | 0.90 | 0.90 | 0.90 | 0.90 | 0.90 | 0.90 | 0.91 | 0.91 | 0.92 | 0.92 | 0.92 | 0.93 | 0.93 | 0.9067 |
|  | MI | 0.89 | 0.90 | 0.90 | 0.90 | 0.90 | 0.90 | 0.90 | 0.90 | 0.90 | 0.90 | 0.90 | 0.90 | 0.90 | 0.91 | 0.91 | 0.92 | 0.92 | 0.92 | 0.93 | 0.93 | 0.9066 |
| 23 | CIM | 0.76 | 0.76 | 0.76 | 0.77 | 0.77 | 0.77 | 0.78 | 0.77 | 0.78 | 0.78 | 0.78 | 0.78 | 0.78 | 0.80 | 0.80 | 0.81 | 0.83 | 0.84 | 0.85 | 0.85 | 0.7925 |
|  | EM | 0.76 | 0.76 | 0.76 | 0.77 | 0.77 | 0.77 | 0.77 | 0.77 | 0.78 | 0.78 | 0.78 | 0.78 | 0.78 | 0.79 | 0.79 | 0.81 | 0.82 | 0.83 | 0.83 | 0.83 | 0.7876 |
|  | MI | 0.76 | 0.76 | 0.76 | 0.77 | 0.77 | 0.77 | 0.78 | 0.77 | 0.78 | 0.78 | 0.78 | 0.78 | 0.78 | 0.79 | 0.79 | 0.81 | 0.82 | 0.83 | 0.83 | 0.83 | 0.7869 |
| 24 | CIM | 0.88 | 0.89 | 0.89 | 0.89 | 0.89 | 0.89 | 0.89 | 0.89 | 0.89 | 0.90 | 0.89 | 0.90 | 0.90 | 0.90 | 0.91 | 0.91 | 0.91 | 0.92 | 0.93 | 0.93 | 0.8998 |
|  | EM | 0.88 | 0.89 | 0.89 | 0.89 | 0.89 | 0.89 | 0.89 | 0.89 | 0.89 | 0.89 | 0.89 | 0.89 | 0.89 | 0.89 | 0.89 | 0.90 | 0.90 | 0.90 | 0.90 | 0.90 | 0.8909 |
|  | MI | 0.88 | 0.89 | 0.89 | 0.89 | 0.89 | 0.89 | 0.89 | 0.89 | 0.89 | 0.89 | 0.89 | 0.89 | 0.89 | 0.89 | 0.89 | 0.90 | 0.90 | 0.90 | 0.90 | 0.90 | 0.8910 |
| 25 | CIM | 0.91 | 0.91 | 0.91 | 0.91 | 0.91 | 0.91 | 0.91 | 0.91 | 0.91 | 0.91 | 0.91 | 0.91 | 0.91 | 0.91 | 0.92 | 0.92 | 0.92 | 0.92 | 0.93 | 0.93 | 0.9146 |
|  | EM | 0.91 | 0.91 | 0.91 | 0.91 | 0.91 | 0.91 | 0.91 | 0.92 | 0.92 | 0.92 | 0.91 | 0.92 | 0.92 | 0.92 | 0.92 | 0.93 | 0.93 | 0.93 | 0.93 | 0.93 | 0.9182 |
|  | MI | 0.91 | 0.91 | 0.91 | 0.91 | 0.91 | 0.91 | 0.91 | 0.92 | 0.92 | 0.91 | 0.91 | 0.92 | 0.92 | 0.92 | 0.92 | 0.93 | 0.93 | 0.93 | 0.93 | 0.93 | 0.9178 |
| 26 | CIM | 0.92 | 0.92 | 0.92 | 0.92 | 0.92 | 0.92 | 0.92 | 0.92 | 0.92 | 0.92 | 0.92 | 0.92 | 0.92 | 0.92 | 0.92 | 0.92 | 0.92 | 0.93 | 0.93 | 0.93 | 0.9208 |
|  | EM | 0.92 | 0.92 | 0.92 | 0.92 | 0.92 | 0.92 | 0.92 | 0.92 | 0.92 | 0.92 | 0.92 | 0.92 | 0.93 | 0.93 | 0.93 | 0.93 | 0.94 | 0.94 | 0.94 | 0.94 | 0.9275 |
|  | MI | 0.92 | 0.92 | 0.92 | 0.92 | 0.92 | 0.92 | 0.92 | 0.92 | 0.92 | 0.92 | 0.92 | 0.93 | 0.93 | 0.93 | 0.93 | 0.93 | 0.93 | 0.94 | 0.94 | 0.95 | 0.9274 |
| 27 | CIM | 0.87 | 0.87 | 0.87 | 0.88 | 0.88 | 0.88 | 0.88 | 0.88 | 0.88 | 0.89 | 0.88 | 0.89 | 0.89 | 0.90 | 0.90 | 0.91 | 0.92 | 0.93 | 0.93 | 0.94 | 0.8938 |
|  | EM | 0.87 | 0.87 | 0.87 | 0.87 | 0.87 | 0.88 | 0.87 | 0.88 | 0.88 | 0.88 | 0.88 | 0.88 | 0.88 | 0.88 | 0.89 | 0.89 | 0.90 | 0.90 | 0.91 | 0.91 | 0.8834 |
|  | MI | 0.87 | 0.87 | 0.87 | 0.87 | 0.87 | 0.88 | 0.88 | 0.88 | 0.88 | 0.88 | 0.88 | 0.88 | 0.88 | 0.88 | 0.89 | 0.89 | 0.90 | 0.90 | 0.91 | 0.91 | 0.8837 |
| 28 | CIM | 0.87 | 0.87 | 0.87 | 0.88 | 0.88 | 0.88 | 0.88 | 0.88 | 0.88 | 0.88 | 0.89 | 0.89 | 0.89 | 0.89 | 0.90 | 0.91 | 0.91 | 0.92 | 0.93 | 0.94 | 0.8928 |
|  | EM | 0.87 | 0.87 | 0.87 | 0.87 | 0.87 | 0.87 | 0.87 | 0.87 | 0.87 | 0.87 | 0.87 | 0.88 | 0.88 | 0.88 | 0.88 | 0.88 | 0.88 | 0.88 | 0.89 | 0.89 | 0.8765 |
|  | MI | 0.87 | 0.87 | 0.87 | 0.87 | 0.87 | 0.87 | 0.88 | 0.87 | 0.87 | 0.87 | 0.87 | 0.88 | 0.88 | 0.88 | 0.88 | 0.88 | 0.88 | 0.88 | 0.89 | 0.89 | 0.8769 |
| 29 | CIM | 0.92 | 0.92 | 0.92 | 0.92 | 0.92 | 0.92 | 0.92 | 0.92 | 0.92 | 0.92 | 0.93 | 0.93 | 0.93 | 0.93 | 0.93 | 0.94 | 0.94 | 0.95 | 0.95 | 0.96 | 0.9301 |
|  | EM | 0.92 | 0.92 | 0.92 | 0.92 | 0.92 | 0.92 | 0.92 | 0.92 | 0.92 | 0.92 | 0.92 | 0.92 | 0.92 | 0.92 | 0.92 | 0.93 | 0.93 | 0.93 | 0.93 | 0.93 | 0.9218 |
|  | MI | 0.92 | 0.92 | 0.92 | 0.92 | 0.92 | 0.92 | 0.92 | 0.92 | 0.92 | 0.92 | 0.92 | 0.92 | 0.92 | 0.92 | 0.92 | 0.93 | 0.93 | 0.93 | 0.93 | 0.93 | 0.9220 |
| 30 | CIM | 0.72 | 0.72 | 0.73 | 0.74 | 0.74 | 0.74 | 0.75 | 0.75 | 0.75 | 0.75 | 0.76 | 0.76 | 0.77 | 0.78 | 0.80 | 0.81 | 0.83 | 0.84 | 0.86 | 0.88 | 0.7768 |
|  | EM | 0.72 | 0.72 | 0.72 | 0.73 | 0.73 | 0.73 | 0.73 | 0.73 | 0.73 | 0.73 | 0.73 | 0.74 | 0.74 | 0.75 | 0.75 | 0.77 | 0.77 | 0.78 | 0.79 | 0.80 | 0.7463 |
|  | MI | 0.72 | 0.72 | 0.72 | 0.73 | 0.73 | 0.73 | 0.75 | 0.73 | 0.73 | 0.73 | 0.73 | 0.74 | 0.74 | 0.75 | 0.75 | 0.76 | 0.77 | 0.78 | 0.79 | 0.80 | 0.7468 |
| 31 | CIM | 0.68 | 0.69 | 0.69 | 0.70 | 0.70 | 0.70 | 0.70 | 0.71 | 0.71 | 0.71 | 0.71 | 0.71 | 0.72 | 0.73 | 0.75 | 0.76 | 0.78 | 0.80 | 0.81 | 0.82 | 0.7311 |
|  | EM | 0.68 | 0.69 | 0.69 | 0.69 | 0.70 | 0.70 | 0.70 | 0.70 | 0.70 | 0.70 | 0.71 | 0.71 | 0.72 | 0.72 | 0.73 | 0.73 | 0.75 | 0.77 | 0.79 | 0.78 | 0.7191 |
|  | MI | 0.68 | 0.69 | 0.69 | 0.69 | 0.70 | 0.70 | 0.70 | 0.70 | 0.70 | 0.70 | 0.71 | 0.70 | 0.72 | 0.72 | 0.73 | 0.73 | 0.75 | 0.77 | 0.78 | 0.78 | 0.7183 |
| 32 | CIM | 0.87 | 0.87 | 0.88 | 0.88 | 0.88 | 0.88 | 0.88 | 0.88 | 0.89 | 0.89 | 0.89 | 0.89 | 0.89 | 0.90 | 0.91 | 0.91 | 0.92 | 0.92 | 0.93 | 0.94 | 0.8964 |
|  | EM | 0.87 | 0.87 | 0.87 | 0.87 | 0.87 | 0.88 | 0.87 | 0.87 | 0.88 | 0.88 | 0.88 | 0.88 | 0.88 | 0.88 | 0.88 | 0.88 | 0.89 | 0.89 | 0.89 | 0.90 | 0.8791 |
|  | MI | 0.87 | 0.87 | 0.87 | 0.87 | 0.87 | 0.88 | 0.88 | 0.87 | 0.88 | 0.88 | 0.88 | 0.88 | 0.88 | 0.88 | 0.88 | 0.88 | 0.89 | 0.89 | 0.89 | 0.90 | 0.8795 |
| 33 | CIM | 0.91 | 0.91 | 0.91 | 0.91 | 0.91 | 0.91 | 0.91 | 0.91 | 0.91 | 0.92 | 0.91 | 0.92 | 0.92 | 0.92 | 0.92 | 0.93 | 0.93 | 0.94 | 0.94 | 0.94 | 0.9199 |
|  | EM | 0.91 | 0.91 | 0.91 | 0.91 | 0.91 | 0.91 | 0.91 | 0.91 | 0.92 | 0.92 | 0.92 | 0.92 | 0.92 | 0.92 | 0.92 | 0.93 | 0.93 | 0.93 | 0.94 | 0.94 | 0.9198 |
|  | MI | 0.91 | 0.91 | 0.91 | 0.91 | 0.91 | 0.91 | 0.91 | 0.91 | 0.92 | 0.92 | 0.92 | 0.92 | 0.92 | 0.92 | 0.92 | 0.93 | 0.93 | 0.93 | 0.94 | 0.94 | 0.9195 |
| 34 | CIM | 0.93 | 0.93 | 0.93 | 0.93 | 0.93 | 0.93 | 0.93 | 0.93 | 0.93 | 0.94 | 0.93 | 0.94 | 0.94 | 0.94 | 0.94 | 0.95 | 0.95 | 0.96 | 0.96 | 0.96 | 0.9395 |
|  | EM | 0.93 | 0.93 | 0.93 | 0.93 | 0.93 | 0.93 | 0.93 | 0.93 | 0.93 | 0.93 | 0.93 | 0.93 | 0.93 | 0.93 | 0.93 | 0.94 | 0.94 | 0.94 | 0.94 | 0.94 | 0.9322 |
|  | MI | 0.93 | 0.93 | 0.93 | 0.93 | 0.93 | 0.93 | 0.93 | 0.93 | 0.93 | 0.93 | 0.93 | 0.93 | 0.93 | 0.93 | 0.93 | 0.93 | 0.94 | 0.94 | 0.94 | 0.94 | 0.9322 |
| 35 | CIM | 0.88 | 0.88 | 0.88 | 0.88 | 0.89 | 0.89 | 0.89 | 0.89 | 0.89 | 0.89 | 0.89 | 0.89 | 0.89 | 0.90 | 0.90 | 0.90 | 0.91 | 0.91 | 0.92 | 0.92 | 0.8942 |
|  | EM | 0.88 | 0.88 | 0.88 | 0.88 | 0.88 | 0.88 | 0.89 | 0.89 | 0.88 | 0.89 | 0.89 | 0.89 | 0.89 | 0.89 | 0.89 | 0.89 | 0.90 | 0.90 | 0.91 | 0.90 | 0.8894 |
|  | MI | 0.88 | 0.88 | 0.88 | 0.88 | 0.88 | 0.88 | 0.89 | 0.89 | 0.88 | 0.89 | 0.89 | 0.89 | 0.89 | 0.89 | 0.89 | 0.89 | 0.90 | 0.90 | 0.91 | 0.90 | 0.8896 |
| 36 | CIM | 0.82 | 0.82 | 0.82 | 0.83 | 0.83 | 0.83 | 0.84 | 0.84 | 0.84 | 0.84 | 0.84 | 0.84 | 0.85 | 0.86 | 0.87 | 0.87 | 0.88 | 0.89 | 0.91 | 0.91 | 0.8533 |
|  | EM | 0.82 | 0.82 | 0.82 | 0.82 | 0.82 | 0.82 | 0.83 | 0.82 | 0.83 | 0.83 | 0.83 | 0.83 | 0.83 | 0.84 | 0.84 | 0.84 | 0.84 | 0.85 | 0.86 | 0.85 | 0.8321 |
|  | MI | 0.82 | 0.82 | 0.82 | 0.82 | 0.82 | 0.82 | 0.84 | 0.82 | 0.83 | 0.83 | 0.83 | 0.83 | 0.83 | 0.84 | 0.84 | 0.84 | 0.84 | 0.85 | 0.85 | 0.85 | 0.8327 |
| 37 | CIM | 0.80 | 0.80 | 0.80 | 0.80 | 0.81 | 0.81 | 0.81 | 0.81 | 0.81 | 0.81 | 0.81 | 0.82 | 0.82 | 0.83 | 0.83 | 0.84 | 0.85 | 0.85 | 0.86 | 0.87 | 0.8228 |
|  | EM | 0.80 | 0.80 | 0.81 | 0.81 | 0.81 | 0.81 | 0.81 | 0.81 | 0.81 | 0.82 | 0.82 | 0.82 | 0.82 | 0.83 | 0.83 | 0.84 | 0.85 | 0.85 | 0.86 | 0.87 | 0.8246 |
|  | MI | 0.80 | 0.80 | 0.80 | 0.81 | 0.81 | 0.81 | 0.81 | 0.81 | 0.81 | 0.82 | 0.82 | 0.82 | 0.82 | 0.83 | 0.83 | 0.84 | 0.85 | 0.85 | 0.86 | 0.87 | 0.8241 |
| 38 | CIM | 0.93 | 0.93 | 0.93 | 0.93 | 0.93 | 0.93 | 0.93 | 0.93 | 0.93 | 0.93 | 0.93 | 0.93 | 0.93 | 0.94 | 0.94 | 0.94 | 0.95 | 0.95 | 0.95 | 0.96 | 0.9364 |
|  | EM | 0.93 | 0.93 | 0.93 | 0.93 | 0.93 | 0.93 | 0.93 | 0.93 | 0.93 | 0.93 | 0.93 | 0.93 | 0.93 | 0.93 | 0.94 | 0.94 | 0.94 | 0.94 | 0.94 | 0.94 | 0.9325 |
|  | MI | 0.93 | 0.93 | 0.93 | 0.93 | 0.93 | 0.93 | 0.93 | 0.93 | 0.93 | 0.93 | 0.93 | 0.93 | 0.93 | 0.93 | 0.94 | 0.94 | 0.94 | 0.94 | 0.94 | 0.95 | 0.9326 |
| 39 | CIM | 0.85 | 0.85 | 0.86 | 0.86 | 0.86 | 0.87 | 0.87 | 0.87 | 0.87 | 0.87 | 0.87 | 0.87 | 0.88 | 0.88 | 0.89 | 0.90 | 0.91 | 0.91 | 0.92 | 0.93 | 0.8808 |
|  | EM | 0.85 | 0.85 | 0.85 | 0.85 | 0.85 | 0.86 | 0.85 | 0.85 | 0.86 | 0.86 | 0.86 | 0.86 | 0.86 | 0.86 | 0.86 | 0.86 | 0.87 | 0.87 | 0.87 | 0.87 | 0.8589 |
|  | MI | 0.85 | 0.85 | 0.85 | 0.85 | 0.85 | 0.86 | 0.87 | 0.85 | 0.86 | 0.86 | 0.86 | 0.86 | 0.86 | 0.86 | 0.86 | 0.86 | 0.87 | 0.87 | 0.87 | 0.88 | 0.8599 |
| 40 | CIM | 0.89 | 0.90 | 0.90 | 0.90 | 0.90 | 0.90 | 0.90 | 0.90 | 0.91 | 0.91 | 0.91 | 0.91 | 0.91 | 0.91 | 0.92 | 0.92 | 0.93 | 0.94 | 0.94 | 0.95 | 0.9129 |
|  | EM | 0.89 | 0.89 | 0.89 | 0.90 | 0.90 | 0.90 | 0.90 | 0.90 | 0.90 | 0.90 | 0.90 | 0.90 | 0.90 | 0.90 | 0.90 | 0.91 | 0.91 | 0.91 | 0.92 | 0.92 | 0.9015 |
|  | MI | 0.89 | 0.89 | 0.89 | 0.90 | 0.90 | 0.90 | 0.90 | 0.90 | 0.90 | 0.90 | 0.90 | 0.90 | 0.90 | 0.90 | 0.90 | 0.90 | 0.91 | 0.91 | 0.92 | 0.92 | 0.9016 |
| 41 | CIM | 0.89 | 0.89 | 0.89 | 0.89 | 0.89 | 0.89 | 0.89 | 0.89 | 0.89 | 0.89 | 0.89 | 0.88 | 0.89 | 0.88 | 0.88 | 0.89 | 0.89 | 0.89 | 0.89 | 0.90 | 0.8866 |
|  | EM | 0.89 | 0.89 | 0.89 | 0.89 | 0.89 | 0.89 | 0.89 | 0.89 | 0.89 | 0.89 | 0.89 | 0.89 | 0.89 | 0.89 | 0.90 | 0.90 | 0.90 | 0.90 | 0.90 | 0.90 | 0.8935 |
|  | MI | 0.89 | 0.89 | 0.89 | 0.89 | 0.89 | 0.89 | 0.89 | 0.89 | 0.89 | 0.89 | 0.89 | 0.89 | 0.89 | 0.89 | 0.90 | 0.90 | 0.90 | 0.90 | 0.90 | 0.90 | 0.8933 |
| 42 | CIM | 0.91 | 0.91 | 0.91 | 0.91 | 0.91 | 0.91 | 0.91 | 0.91 | 0.91 | 0.91 | 0.91 | 0.91 | 0.91 | 0.92 | 0.92 | 0.92 | 0.93 | 0.93 | 0.93 | 0.94 | 0.9151 |
|  | EM | 0.91 | 0.91 | 0.91 | 0.91 | 0.91 | 0.91 | 0.91 | 0.91 | 0.91 | 0.91 | 0.91 | 0.91 | 0.91 | 0.91 | 0.92 | 0.92 | 0.92 | 0.92 | 0.93 | 0.93 | 0.9138 |
|  | MI | 0.91 | 0.91 | 0.91 | 0.91 | 0.91 | 0.91 | 0.91 | 0.91 | 0.91 | 0.91 | 0.91 | 0.91 | 0.91 | 0.91 | 0.92 | 0.92 | 0.92 | 0.92 | 0.93 | 0.93 | 0.9137 |
| 43 | CIM | 0.88 | 0.88 | 0.88 | 0.88 | 0.88 | 0.88 | 0.88 | 0.89 | 0.89 | 0.88 | 0.88 | 0.88 | 0.89 | 0.89 | 0.89 | 0.89 | 0.89 | 0.90 | 0.90 | 0.91 | 0.8875 |
|  | EM | 0.88 | 0.88 | 0.89 | 0.89 | 0.89 | 0.89 | 0.89 | 0.89 | 0.89 | 0.89 | 0.89 | 0.89 | 0.89 | 0.90 | 0.90 | 0.90 | 0.91 | 0.92 | 0.92 | 0.92 | 0.8969 |
|  | MI | 0.88 | 0.88 | 0.89 | 0.89 | 0.89 | 0.89 | 0.88 | 0.89 | 0.89 | 0.89 | 0.89 | 0.89 | 0.89 | 0.90 | 0.90 | 0.90 | 0.91 | 0.92 | 0.92 | 0.92 | 0.8965 |
| 44 | CIM | 0.88 | 0.88 | 0.88 | 0.88 | 0.88 | 0.88 | 0.88 | 0.88 | 0.88 | 0.88 | 0.88 | 0.88 | 0.88 | 0.88 | 0.88 | 0.88 | 0.89 | 0.89 | 0.89 | 0.89 | 0.8802 |
|  | EM | 0.88 | 0.88 | 0.88 | 0.88 | 0.88 | 0.88 | 0.88 | 0.88 | 0.88 | 0.88 | 0.88 | 0.88 | 0.88 | 0.88 | 0.89 | 0.89 | 0.89 | 0.89 | 0.90 | 0.89 | 0.8841 |
|  | MI | 0.88 | 0.88 | 0.88 | 0.88 | 0.88 | 0.88 | 0.88 | 0.88 | 0.88 | 0.88 | 0.88 | 0.88 | 0.88 | 0.88 | 0.89 | 0.89 | 0.89 | 0.89 | 0.90 | 0.90 | 0.8842 |
| 45 | CIM | 0.89 | 0.89 | 0.89 | 0.89 | 0.90 | 0.90 | 0.90 | 0.90 | 0.90 | 0.90 | 0.90 | 0.90 | 0.91 | 0.91 | 0.92 | 0.92 | 0.93 | 0.93 | 0.94 | 0.94 | 0.9079 |
|  | EM | 0.89 | 0.89 | 0.89 | 0.89 | 0.89 | 0.89 | 0.89 | 0.89 | 0.89 | 0.89 | 0.89 | 0.89 | 0.89 | 0.89 | 0.89 | 0.90 | 0.90 | 0.90 | 0.90 | 0.90 | 0.8917 |
|  | MI | 0.89 | 0.89 | 0.89 | 0.89 | 0.89 | 0.89 | 0.90 | 0.89 | 0.89 | 0.89 | 0.89 | 0.89 | 0.89 | 0.89 | 0.89 | 0.90 | 0.90 | 0.90 | 0.90 | 0.91 | 0.8925 |
| 46 | CIM | 0.89 | 0.89 | 0.90 | 0.89 | 0.90 | 0.90 | 0.90 | 0.90 | 0.90 | 0.90 | 0.90 | 0.90 | 0.90 | 0.90 | 0.90 | 0.90 | 0.90 | 0.91 | 0.91 | 0.91 | 0.8987 |
|  | EM | 0.89 | 0.90 | 0.90 | 0.90 | 0.90 | 0.90 | 0.90 | 0.90 | 0.90 | 0.90 | 0.90 | 0.90 | 0.90 | 0.91 | 0.91 | 0.91 | 0.92 | 0.92 | 0.93 | 0.93 | 0.9070 |
|  | MI | 0.89 | 0.90 | 0.90 | 0.90 | 0.90 | 0.90 | 0.90 | 0.90 | 0.90 | 0.90 | 0.90 | 0.90 | 0.90 | 0.91 | 0.91 | 0.91 | 0.92 | 0.92 | 0.93 | 0.93 | 0.9066 |
| 47 | CIM | 0.57 | 0.57 | 0.58 | 0.59 | 0.59 | 0.60 | 0.59 | 0.60 | 0.60 | 0.61 | 0.61 | 0.61 | 0.61 | 0.64 | 0.66 | 0.68 | 0.69 | 0.72 | 0.74 | 0.75 | 0.6337 |
|  | EM | 0.57 | 0.57 | 0.58 | 0.58 | 0.58 | 0.58 | 0.58 | 0.59 | 0.59 | 0.59 | 0.59 | 0.59 | 0.59 | 0.61 | 0.62 | 0.63 | 0.64 | 0.67 | 0.67 | 0.67 | 0.6061 |
|  | MI | 0.57 | 0.57 | 0.58 | 0.58 | 0.58 | 0.58 | 0.59 | 0.59 | 0.59 | 0.60 | 0.59 | 0.59 | 0.59 | 0.61 | 0.62 | 0.63 | 0.64 | 0.67 | 0.67 | 0.67 | 0.6069 |
| 48 | CIM | 0.81 | 0.81 | 0.81 | 0.82 | 0.82 | 0.82 | 0.82 | 0.83 | 0.83 | 0.83 | 0.83 | 0.83 | 0.84 | 0.84 | 0.86 | 0.87 | 0.88 | 0.88 | 0.90 | 0.90 | 0.8416 |
|  | EM | 0.81 | 0.81 | 0.81 | 0.81 | 0.81 | 0.81 | 0.81 | 0.81 | 0.82 | 0.82 | 0.81 | 0.82 | 0.82 | 0.82 | 0.82 | 0.83 | 0.84 | 0.84 | 0.84 | 0.84 | 0.8201 |
|  | MI | 0.81 | 0.81 | 0.81 | 0.81 | 0.81 | 0.81 | 0.82 | 0.81 | 0.82 | 0.81 | 0.81 | 0.82 | 0.82 | 0.82 | 0.82 | 0.83 | 0.84 | 0.84 | 0.84 | 0.84 | 0.8204 |
| 49 | CIM | 0.81 | 0.81 | 0.82 | 0.82 | 0.83 | 0.83 | 0.83 | 0.83 | 0.83 | 0.84 | 0.84 | 0.84 | 0.84 | 0.85 | 0.86 | 0.87 | 0.89 | 0.89 | 0.90 | 0.92 | 0.8491 |
|  | EM | 0.81 | 0.81 | 0.82 | 0.82 | 0.82 | 0.82 | 0.82 | 0.82 | 0.82 | 0.82 | 0.83 | 0.83 | 0.83 | 0.83 | 0.84 | 0.84 | 0.85 | 0.85 | 0.86 | 0.86 | 0.8299 |
|  | MI | 0.81 | 0.81 | 0.82 | 0.82 | 0.82 | 0.82 | 0.83 | 0.82 | 0.82 | 0.82 | 0.83 | 0.83 | 0.82 | 0.83 | 0.84 | 0.84 | 0.85 | 0.85 | 0.86 | 0.87 | 0.8301 |
| 50 | CIM | 0.83 | 0.83 | 0.83 | 0.83 | 0.83 | 0.83 | 0.83 | 0.83 | 0.83 | 0.83 | 0.83 | 0.84 | 0.83 | 0.84 | 0.83 | 0.84 | 0.85 | 0.85 | 0.85 | 0.86 | 0.8375 |
|  | EM | 0.83 | 0.83 | 0.83 | 0.84 | 0.84 | 0.84 | 0.84 | 0.84 | 0.84 | 0.84 | 0.84 | 0.85 | 0.85 | 0.85 | 0.85 | 0.86 | 0.87 | 0.88 | 0.87 | 0.88 | 0.8487 |
|  | MI | 0.83 | 0.83 | 0.83 | 0.84 | 0.84 | 0.84 | 0.83 | 0.84 | 0.84 | 0.84 | 0.84 | 0.85 | 0.85 | 0.85 | 0.85 | 0.86 | 0.87 | 0.88 | 0.87 | 0.88 | 0.8484 |
| **WHOLE** | CIM | 0.85 | 0.85 | 0.85 | 0.85 | 0.86 | 0.86 | 0.86 | 0.86 | 0.86 | 0.86 | 0.86 | 0.86 | 0.87 | 0.87 | 0.88 | 0.88 | 0.89 | 0.90 | 0.91 | 0.91 | **0.87054** |
|  | EM | 0.85 | 0.85 | 0.85 | 0.85 | 0.85 | 0.85 | 0.85 | 0.86 | 0.86 | 0.86 | 0.86 | 0.86 | 0.86 | 0.86 | 0.87 | 0.87 | 0.88 | 0.88 | 0.88 | 0.89 | **0.86244** |
|  | MI | 0.85 | 0.85 | 0.85 | 0.85 | 0.85 | 0.85 | 0.86 | 0.86 | 0.86 | 0.86 | 0.86 | 0.86 | 0.86 | 0.86 | 0.87 | 0.87 | 0.87 | 0.88 | 0.88 | 0.89 | **0.86252** |
