# Supplementary material for: The impact of missing data rates and imputation methods on the assumption of unidimensionality
Source: PLoS One. 2025 Apr 30;20(4):e0321344. doi: 10.1371/journal.pone.0321344 (PMC12043241; doi:10.1371/journal.pone.0321344)
Supplement: Appendix 2 — (DOCX) [file pone.0321344.s002.docx]

Appendix (2)

Eigenvalues of the first three components in detail for each imputation method.

|  | **CIM** | | | **EM** | | | **MI** | | |
| --- | --- | --- | --- | --- | --- | --- | --- | --- | --- |
|  | **1** | **2** | **3** | **1** | **2** | **3** | **1** | **2** | **3** |
| 0% | 36.78 | 1.88 | 0.77 | 36.78 | 1.88 | 0.77 | 36.78 | 1.88 | 0.77 |
| 2% | 36.91 | 1.84 | 0.75 | 36.90 | 1.89 | 0.77 | 36.90 | 1.89 | 0.77 |
| 4% | 37.04 | 1.80 | 0.73 | 37.02 | 1.89 | 0.77 | 37.02 | 1.89 | 0.77 |
| 6% | 37.16 | 1.74 | 0.70 | 37.13 | 1.89 | 0.77 | 37.12 | 1.89 | 0.77 |
| 7% | 37.22 | 1.72 | 0.70 | 37.18 | 1.90 | 0.77 | 37.18 | 1.90 | 0.77 |
| 8% | 37.30 | 1.70 | 0.68 | 37.25 | 1.89 | 0.77 | 37.25 | 1.89 | 0.77 |
| 9% | 37.34 | 1.67 | 0.69 | 37.29 | 1.90 | 0.78 | 37.28 | 1.89 | 0.78 |
| 10% | 37.43 | 1.66 | 0.67 | 37.37 | 1.90 | 0.78 | 37.36 | 1.90 | 0.78 |
| 11% | 37.48 | 1.64 | 0.66 | 37.40 | 1.91 | 0.77 | 37.39 | 1.91 | 0.77 |
| 12% | 37.57 | 1.61 | 0.65 | 37.49 | 1.90 | 0.78 | 37.48 | 1.90 | 0.78 |
| 13% | 37.59 | 1.59 | 0.65 | 37.50 | 1.91 | 0.78 | 37.49 | 1.91 | 0.78 |
| 14% | 37.68 | 1.58 | 0.65 | 37.58 | 1.91 | 0.79 | 37.58 | 1.92 | 0.79 |
| 15% | 37.78 | 1.55 | 0.64 | 37.66 | 1.90 | 0.78 | 37.66 | 1.90 | 0.78 |
| 20% | 38.12 | 1.45 | 0.60 | 37.99 | 1.91 | 0.79 | 37.97 | 1.92 | 0.79 |
| 25% | 38.45 | 1.35 | 0.58 | 38.25 | 1.92 | 0.79 | 38.23 | 1.93 | 0.79 |
| 30% | 38.83 | 1.27 | 0.55 | 38.55 | 1.94 | 0.77 | 38.53 | 1.94 | 0.77 |
| 35% | 39.28 | 1.15 | 0.53 | 38.94 | 1.92 | 0.78 | 38.91 | 1.92 | 0.77 |
| 40% | 39.77 | 1.07 | 0.49 | 39.36 | 1.92 | 0.77 | 39.33 | 1.92 | 0.77 |
| 45% | 40.16 | 1.00 | 0.48 | 39.66 | 1.94 | 0.78 | 39.62 | 1.95 | 0.77 |
| 50% | 40.56 | 0.93 | 0.45 | 39.83 | 1.92 | 0.75 | 39.92 | 1.92 | 0.75 |
| **MEAN** | **38.088** | **1.491** | **0.623** | **37.913** | **1.909** | **0.776** | **37.905** | **1.910** | **0.775** |

**First Index due to Method of imputing missingness**

| **%** | **CIM** | **EM** | **MI** |
| --- | --- | --- | --- |
| 0% | 19.55 | 19.55 | 19.55 |
| 2% | 20.06 | 19.53 | 19.54 |
| 4% | 20.62 | 19.59 | 19.59 |
| 6% | 21.33 | 19.64 | 19.63 |
| 7% | 21.60 | 19.59 | 19.60 |
| 8% | 21.95 | 19.68 | 19.68 |
| 9% | 22.33 | 19.67 | 19.69 |
| 10% | 22.57 | 19.65 | 19.65 |
| 11% | 22.83 | 19.60 | 19.62 |
| 12% | 23.29 | 19.71 | 19.71 |
| 13% | 23.58 | 19.62 | 19.61 |
| 14% | 23.84 | 19.63 | 19.62 |
| 15% | 24.40 | 19.77 | 19.79 |
| 20% | 26.33 | 19.88 | 19.83 |
| 25% | 28.40 | 19.88 | 19.84 |
| 30% | 30.63 | 19.84 | 19.86 |
| 35% | 34.13 | 20.28 | 20.26 |
| 40% | 37.21 | 20.53 | 20.52 |
| 45% | 40.27 | 20.40 | 20.27 |
| 50% | 43.72 | 20.74 | 20.76 |
| MEAN | 26.43 | 19.84 | 19.83 |
| Formula for First Index is $\left( First Eigenvalue \right)/\left( Second Eigenvalue \right)$ | | | |

**Second Index due to Method of imputing missingness**

| **%** | **CIM** | **EM** | **MI** |
| --- | --- | --- | --- |
| 0% | 31.44 | 31.44 | 31.44 |
| 2% | 32.14 | 31.38 | 31.42 |
| 4% | 33.04 | 31.44 | 31.44 |
| 6% | 34.07 | 31.43 | 31.41 |
| 7% | 34.58 | 31.28 | 31.28 |
| 8% | 35.03 | 31.41 | 31.43 |
| 9% | 36.15 | 31.69 | 31.73 |
| 10% | 36.09 | 31.60 | 31.62 |
| 11% | 36.51 | 31.25 | 31.27 |
| 12% | 37.34 | 31.71 | 31.65 |
| 13% | 37.99 | 31.47 | 31.45 |
| 14% | 38.61 | 31.67 | 31.60 |
| 15% | 39.93 | 31.77 | 31.79 |
| 20% | 43.23 | 32.14 | 31.97 |
| 25% | 47.67 | 31.93 | 31.89 |
| 30% | 52.16 | 31.29 | 31.29 |
| 35% | 60.93 | 32.44 | 32.23 |
| 40% | 66.85 | 32.72 | 32.51 |
| 45% | 75.28 | 32.30 | 31.93 |
| 50% | 83.83 | 32.52 | 32.47 |
| MEAN | 44.64 | 31.74 | 31.69 |
| Formula for Second Index is $\left( First-Second \right)/\left( Second-Third \right)$ | | | |
