# Supplementary material for: The impact of missing data rates and imputation methods on the assumption of unidimensionality
Source: PLoS One. 2025 Apr 30;20(4):e0321344. doi: 10.1371/journal.pone.0321344 (PMC12043241; doi:10.1371/journal.pone.0321344)
Supplement: Appendix 3 — (DOCX) [file pone.0321344.s003.docx]

Appendix (3)

Communalities due to Percentages of Missingness through CIM, EM, and MI imputation methods

| **ITEM** | **METHOD** | **0%** | **2%** | **4%** | **6%** | **7%** | **8%** | **9%** | **10%** | **11%** | **12%** | **13%** | **14%** | **15%** | **20%** | **25%** | **30%** | **35%** | **40%** | **45%** | **50%** | **MEAN** |
| --- | --- | --- | --- | --- | --- | --- | --- | --- | --- | --- | --- | --- | --- | --- | --- | --- | --- | --- | --- | --- | --- | --- |
| 1 | CIM | 0.81 | 0.82 | 0.81 | 0.82 | 0.82 | 0.81 | 0.81 | 0.81 | 0.81 | 0.82 | 0.82 | 0.81 | 0.82 | 0.82 | 0.82 | 0.83 | 0.82 | 0.83 | 0.70 | 0.72 | 0.8058 |
|  | EM | 0.81 | 0.82 | 0.82 | 0.82 | 0.83 | 0.83 | 0.83 | 0.83 | 0.83 | 0.83 | 0.83 | 0.83 | 0.84 | 0.84 | 0.85 | 0.86 | 0.86 | 0.87 | 0.87 | 0.90 | 0.8422 |
|  | MI | 0.81 | 0.82 | 0.82 | 0.82 | 0.83 | 0.83 | 0.83 | 0.83 | 0.83 | 0.83 | 0.83 | 0.83 | 0.84 | 0.84 | 0.85 | 0.86 | 0.86 | 0.87 | 0.87 | 0.90 | 0.8418 |
| 2 | CIM | 0.78 | 0.78 | 0.78 | 0.79 | 0.78 | 0.79 | 0.79 | 0.79 | 0.79 | 0.79 | 0.79 | 0.79 | 0.80 | 0.80 | 0.80 | 0.82 | 0.83 | 0.83 | 0.83 | 0.84 | 0.8008 |
|  | EM | 0.78 | 0.78 | 0.78 | 0.79 | 0.78 | 0.79 | 0.79 | 0.79 | 0.79 | 0.79 | 0.79 | 0.79 | 0.79 | 0.80 | 0.80 | 0.81 | 0.82 | 0.82 | 0.82 | 0.83 | 0.7954 |
|  | MI | 0.78 | 0.78 | 0.78 | 0.79 | 0.78 | 0.79 | 0.79 | 0.79 | 0.79 | 0.79 | 0.79 | 0.79 | 0.79 | 0.80 | 0.80 | 0.81 | 0.82 | 0.82 | 0.82 | 0.83 | 0.7952 |
| 3 | CIM | 0.80 | 0.80 | 0.80 | 0.80 | 0.80 | 0.81 | 0.81 | 0.81 | 0.81 | 0.81 | 0.81 | 0.81 | 0.81 | 0.82 | 0.82 | 0.83 | 0.84 | 0.85 | 0.85 | 0.86 | 0.8175 |
|  | EM | 0.80 | 0.80 | 0.80 | 0.80 | 0.80 | 0.81 | 0.81 | 0.81 | 0.81 | 0.81 | 0.81 | 0.82 | 0.82 | 0.82 | 0.83 | 0.84 | 0.84 | 0.86 | 0.86 | 0.87 | 0.8223 |
|  | MI | 0.80 | 0.80 | 0.80 | 0.80 | 0.80 | 0.81 | 0.81 | 0.81 | 0.81 | 0.81 | 0.81 | 0.82 | 0.82 | 0.82 | 0.83 | 0.84 | 0.84 | 0.86 | 0.86 | 0.87 | 0.8220 |
| 4 | CIM | 0.80 | 0.80 | 0.81 | 0.81 | 0.81 | 0.81 | 0.81 | 0.81 | 0.81 | 0.81 | 0.81 | 0.81 | 0.81 | 0.81 | 0.82 | 0.82 | 0.83 | 0.84 | 0.84 | 0.84 | 0.8164 |
|  | EM | 0.80 | 0.81 | 0.81 | 0.81 | 0.81 | 0.81 | 0.81 | 0.82 | 0.82 | 0.82 | 0.82 | 0.82 | 0.82 | 0.83 | 0.84 | 0.84 | 0.85 | 0.86 | 0.87 | 0.87 | 0.8285 |
|  | MI | 0.80 | 0.81 | 0.81 | 0.81 | 0.81 | 0.81 | 0.81 | 0.82 | 0.82 | 0.82 | 0.82 | 0.82 | 0.82 | 0.83 | 0.83 | 0.84 | 0.85 | 0.86 | 0.87 | 0.87 | 0.8285 |
| 5 | CIM | 0.75 | 0.75 | 0.76 | 0.76 | 0.76 | 0.76 | 0.77 | 0.77 | 0.77 | 0.77 | 0.77 | 0.77 | 0.77 | 0.78 | 0.79 | 0.80 | 0.81 | 0.82 | 0.82 | 0.83 | 0.7801 |
|  | EM | 0.75 | 0.75 | 0.76 | 0.76 | 0.76 | 0.76 | 0.77 | 0.77 | 0.77 | 0.77 | 0.77 | 0.77 | 0.77 | 0.78 | 0.79 | 0.80 | 0.81 | 0.82 | 0.83 | 0.84 | 0.7812 |
|  | MI | 0.75 | 0.75 | 0.76 | 0.76 | 0.76 | 0.76 | 0.77 | 0.77 | 0.77 | 0.77 | 0.77 | 0.77 | 0.77 | 0.78 | 0.79 | 0.79 | 0.81 | 0.82 | 0.83 | 0.84 | 0.7808 |
| 6 | CIM | 0.82 | 0.82 | 0.82 | 0.83 | 0.83 | 0.83 | 0.83 | 0.83 | 0.83 | 0.83 | 0.83 | 0.83 | 0.84 | 0.84 | 0.84 | 0.85 | 0.85 | 0.86 | 0.86 | 0.87 | 0.8376 |
|  | EM | 0.82 | 0.82 | 0.82 | 0.83 | 0.83 | 0.83 | 0.83 | 0.83 | 0.83 | 0.83 | 0.83 | 0.83 | 0.83 | 0.84 | 0.84 | 0.84 | 0.84 | 0.85 | 0.85 | 0.85 | 0.8333 |
|  | MI | 0.82 | 0.82 | 0.82 | 0.83 | 0.83 | 0.83 | 0.83 | 0.83 | 0.83 | 0.83 | 0.83 | 0.83 | 0.83 | 0.84 | 0.84 | 0.84 | 0.84 | 0.85 | 0.85 | 0.85 | 0.8338 |
| 7 | CIM | 0.72 | 0.73 | 0.73 | 0.73 | 0.73 | 0.73 | 0.74 | 0.74 | 0.74 | 0.74 | 0.74 | 0.74 | 0.75 | 0.75 | 0.76 | 0.78 | 0.78 | 0.80 | 0.81 | 0.82 | 0.7549 |
|  | EM | 0.72 | 0.73 | 0.73 | 0.73 | 0.74 | 0.74 | 0.74 | 0.74 | 0.74 | 0.74 | 0.74 | 0.75 | 0.75 | 0.75 | 0.76 | 0.78 | 0.78 | 0.80 | 0.81 | 0.81 | 0.7554 |
|  | MI | 0.72 | 0.73 | 0.73 | 0.73 | 0.73 | 0.74 | 0.74 | 0.74 | 0.74 | 0.74 | 0.74 | 0.75 | 0.75 | 0.75 | 0.76 | 0.78 | 0.78 | 0.80 | 0.81 | 0.81 | 0.7549 |
| 8 | CIM | 0.46 | 0.47 | 0.47 | 0.48 | 0.49 | 0.49 | 0.49 | 0.51 | 0.49 | 0.51 | 0.51 | 0.52 | 0.52 | 0.53 | 0.56 | 0.58 | 0.59 | 0.61 | 0.65 | 0.68 | 0.5337 |
|  | EM | 0.46 | 0.46 | 0.46 | 0.48 | 0.48 | 0.48 | 0.48 | 0.49 | 0.48 | 0.49 | 0.49 | 0.49 | 0.50 | 0.51 | 0.53 | 0.54 | 0.54 | 0.56 | 0.59 | 0.60 | 0.5087 |
|  | MI | 0.46 | 0.46 | 0.47 | 0.48 | 0.47 | 0.48 | 0.48 | 0.49 | 0.48 | 0.49 | 0.49 | 0.50 | 0.50 | 0.51 | 0.53 | 0.54 | 0.54 | 0.56 | 0.59 | 0.60 | 0.5087 |
| 9 | CIM | 0.85 | 0.85 | 0.85 | 0.85 | 0.84 | 0.85 | 0.84 | 0.84 | 0.84 | 0.84 | 0.84 | 0.84 | 0.84 | 0.84 | 0.83 | 0.83 | 0.83 | 0.83 | 0.72 | 0.73 | 0.8274 |
|  | EM | 0.85 | 0.85 | 0.86 | 0.85 | 0.86 | 0.86 | 0.86 | 0.86 | 0.86 | 0.86 | 0.86 | 0.86 | 0.86 | 0.87 | 0.86 | 0.87 | 0.88 | 0.88 | 0.89 | 0.88 | 0.8637 |
|  | MI | 0.85 | 0.85 | 0.86 | 0.85 | 0.86 | 0.86 | 0.86 | 0.86 | 0.86 | 0.86 | 0.86 | 0.86 | 0.86 | 0.87 | 0.86 | 0.87 | 0.88 | 0.88 | 0.89 | 0.89 | 0.8641 |
| 10 | CIM | 0.70 | 0.70 | 0.71 | 0.71 | 0.71 | 0.71 | 0.71 | 0.71 | 0.72 | 0.72 | 0.72 | 0.72 | 0.72 | 0.73 | 0.74 | 0.75 | 0.76 | 0.77 | 0.78 | 0.79 | 0.7289 |
|  | EM | 0.70 | 0.70 | 0.71 | 0.71 | 0.71 | 0.71 | 0.71 | 0.71 | 0.72 | 0.72 | 0.72 | 0.72 | 0.73 | 0.74 | 0.75 | 0.76 | 0.77 | 0.78 | 0.80 | 0.81 | 0.7372 |
|  | MI | 0.70 | 0.70 | 0.71 | 0.71 | 0.71 | 0.71 | 0.71 | 0.71 | 0.72 | 0.72 | 0.72 | 0.72 | 0.72 | 0.74 | 0.75 | 0.76 | 0.77 | 0.78 | 0.80 | 0.81 | 0.7366 |
| 11 | CIM | 0.72 | 0.72 | 0.72 | 0.72 | 0.73 | 0.73 | 0.73 | 0.73 | 0.73 | 0.73 | 0.73 | 0.74 | 0.74 | 0.75 | 0.75 | 0.75 | 0.76 | 0.77 | 0.75 | 0.76 | 0.7389 |
|  | EM | 0.72 | 0.72 | 0.72 | 0.73 | 0.73 | 0.74 | 0.74 | 0.74 | 0.74 | 0.75 | 0.74 | 0.75 | 0.75 | 0.76 | 0.77 | 0.78 | 0.78 | 0.80 | 0.81 | 0.81 | 0.7542 |
|  | MI | 0.72 | 0.72 | 0.72 | 0.73 | 0.73 | 0.74 | 0.74 | 0.74 | 0.74 | 0.74 | 0.74 | 0.75 | 0.75 | 0.76 | 0.77 | 0.78 | 0.78 | 0.80 | 0.80 | 0.80 | 0.7536 |
| 12 | CIM | 0.82 | 0.83 | 0.82 | 0.82 | 0.82 | 0.82 | 0.82 | 0.82 | 0.82 | 0.82 | 0.82 | 0.82 | 0.82 | 0.82 | 0.82 | 0.82 | 0.82 | 0.82 | 0.81 | 0.82 | 0.8204 |
|  | EM | 0.82 | 0.83 | 0.83 | 0.83 | 0.83 | 0.83 | 0.83 | 0.83 | 0.83 | 0.83 | 0.83 | 0.83 | 0.83 | 0.84 | 0.84 | 0.85 | 0.85 | 0.86 | 0.86 | 0.87 | 0.8396 |
|  | MI | 0.82 | 0.83 | 0.83 | 0.83 | 0.83 | 0.83 | 0.83 | 0.83 | 0.83 | 0.83 | 0.83 | 0.83 | 0.83 | 0.84 | 0.84 | 0.85 | 0.85 | 0.86 | 0.86 | 0.87 | 0.8396 |
| 13 | CIM | 0.82 | 0.82 | 0.82 | 0.82 | 0.82 | 0.82 | 0.82 | 0.82 | 0.82 | 0.82 | 0.82 | 0.82 | 0.82 | 0.83 | 0.83 | 0.84 | 0.84 | 0.85 | 0.81 | 0.83 | 0.8242 |
|  | EM | 0.82 | 0.82 | 0.82 | 0.83 | 0.83 | 0.83 | 0.83 | 0.83 | 0.83 | 0.83 | 0.84 | 0.83 | 0.84 | 0.84 | 0.85 | 0.86 | 0.86 | 0.87 | 0.87 | 0.88 | 0.8415 |
|  | MI | 0.82 | 0.82 | 0.82 | 0.83 | 0.83 | 0.83 | 0.83 | 0.83 | 0.83 | 0.83 | 0.84 | 0.83 | 0.84 | 0.84 | 0.85 | 0.86 | 0.86 | 0.87 | 0.87 | 0.88 | 0.8413 |
| 14 | CIM | 0.86 | 0.86 | 0.86 | 0.86 | 0.86 | 0.86 | 0.86 | 0.86 | 0.86 | 0.86 | 0.86 | 0.86 | 0.86 | 0.86 | 0.86 | 0.87 | 0.87 | 0.87 | 0.87 | 0.88 | 0.8628 |
|  | EM | 0.86 | 0.86 | 0.86 | 0.86 | 0.86 | 0.86 | 0.87 | 0.86 | 0.87 | 0.87 | 0.87 | 0.87 | 0.87 | 0.88 | 0.88 | 0.88 | 0.88 | 0.89 | 0.89 | 0.90 | 0.8726 |
|  | MI | 0.86 | 0.86 | 0.86 | 0.86 | 0.86 | 0.86 | 0.87 | 0.86 | 0.87 | 0.87 | 0.87 | 0.87 | 0.87 | 0.87 | 0.88 | 0.88 | 0.88 | 0.89 | 0.89 | 0.90 | 0.8725 |
| 15 | CIM | 0.86 | 0.86 | 0.86 | 0.86 | 0.86 | 0.86 | 0.86 | 0.86 | 0.86 | 0.87 | 0.86 | 0.86 | 0.86 | 0.87 | 0.87 | 0.87 | 0.88 | 0.88 | 0.83 | 0.84 | 0.8623 |
|  | EM | 0.86 | 0.86 | 0.86 | 0.86 | 0.87 | 0.87 | 0.87 | 0.87 | 0.87 | 0.87 | 0.87 | 0.87 | 0.88 | 0.88 | 0.89 | 0.89 | 0.90 | 0.91 | 0.90 | 0.91 | 0.8794 |
|  | MI | 0.86 | 0.86 | 0.86 | 0.86 | 0.87 | 0.87 | 0.87 | 0.87 | 0.87 | 0.87 | 0.87 | 0.87 | 0.88 | 0.88 | 0.89 | 0.89 | 0.90 | 0.91 | 0.90 | 0.91 | 0.8792 |
| 16 | CIM | 0.69 | 0.70 | 0.70 | 0.71 | 0.71 | 0.71 | 0.71 | 0.72 | 0.71 | 0.72 | 0.73 | 0.72 | 0.72 | 0.74 | 0.75 | 0.75 | 0.77 | 0.79 | 0.79 | 0.81 | 0.7349 |
|  | EM | 0.69 | 0.70 | 0.70 | 0.70 | 0.70 | 0.70 | 0.71 | 0.71 | 0.71 | 0.72 | 0.72 | 0.72 | 0.71 | 0.72 | 0.73 | 0.74 | 0.75 | 0.77 | 0.77 | 0.77 | 0.7237 |
|  | MI | 0.69 | 0.70 | 0.70 | 0.70 | 0.70 | 0.70 | 0.71 | 0.71 | 0.71 | 0.72 | 0.72 | 0.72 | 0.71 | 0.72 | 0.73 | 0.74 | 0.75 | 0.77 | 0.77 | 0.78 | 0.7233 |
| 17 | CIM | 0.91 | 0.91 | 0.91 | 0.91 | 0.91 | 0.91 | 0.91 | 0.91 | 0.91 | 0.91 | 0.91 | 0.91 | 0.91 | 0.91 | 0.91 | 0.91 | 0.91 | 0.91 | 0.91 | 0.91 | 0.9074 |
|  | EM | 0.91 | 0.91 | 0.91 | 0.91 | 0.91 | 0.91 | 0.91 | 0.91 | 0.91 | 0.91 | 0.91 | 0.91 | 0.91 | 0.92 | 0.92 | 0.92 | 0.92 | 0.92 | 0.93 | 0.93 | 0.9154 |
|  | MI | 0.91 | 0.91 | 0.91 | 0.91 | 0.91 | 0.91 | 0.91 | 0.91 | 0.91 | 0.91 | 0.91 | 0.91 | 0.91 | 0.92 | 0.92 | 0.92 | 0.92 | 0.92 | 0.93 | 0.93 | 0.9153 |
| 18 | CIM | 0.85 | 0.85 | 0.85 | 0.85 | 0.85 | 0.85 | 0.84 | 0.85 | 0.85 | 0.84 | 0.84 | 0.84 | 0.84 | 0.83 | 0.83 | 0.83 | 0.83 | 0.83 | 0.80 | 0.81 | 0.8373 |
|  | EM | 0.85 | 0.86 | 0.86 | 0.86 | 0.86 | 0.86 | 0.86 | 0.86 | 0.86 | 0.86 | 0.87 | 0.87 | 0.87 | 0.87 | 0.88 | 0.88 | 0.89 | 0.89 | 0.89 | 0.89 | 0.8700 |
|  | MI | 0.85 | 0.86 | 0.86 | 0.86 | 0.86 | 0.86 | 0.86 | 0.86 | 0.86 | 0.86 | 0.87 | 0.86 | 0.87 | 0.87 | 0.88 | 0.88 | 0.89 | 0.89 | 0.89 | 0.89 | 0.8697 |
| 19 | CIM | 0.59 | 0.59 | 0.60 | 0.61 | 0.62 | 0.61 | 0.62 | 0.63 | 0.62 | 0.63 | 0.63 | 0.64 | 0.65 | 0.65 | 0.67 | 0.70 | 0.72 | 0.74 | 0.76 | 0.77 | 0.6550 |
|  | EM | 0.59 | 0.59 | 0.60 | 0.60 | 0.60 | 0.60 | 0.60 | 0.61 | 0.60 | 0.61 | 0.61 | 0.62 | 0.62 | 0.62 | 0.63 | 0.64 | 0.66 | 0.67 | 0.69 | 0.69 | 0.6241 |
|  | MI | 0.59 | 0.59 | 0.60 | 0.60 | 0.60 | 0.60 | 0.60 | 0.61 | 0.60 | 0.61 | 0.61 | 0.62 | 0.62 | 0.62 | 0.63 | 0.64 | 0.65 | 0.67 | 0.69 | 0.69 | 0.6237 |
| 20 | CIM | 0.78 | 0.78 | 0.78 | 0.79 | 0.79 | 0.79 | 0.79 | 0.79 | 0.79 | 0.79 | 0.79 | 0.79 | 0.79 | 0.80 | 0.80 | 0.81 | 0.81 | 0.82 | 0.81 | 0.82 | 0.7968 |
|  | EM | 0.78 | 0.78 | 0.79 | 0.79 | 0.79 | 0.79 | 0.79 | 0.79 | 0.80 | 0.79 | 0.80 | 0.80 | 0.80 | 0.81 | 0.81 | 0.82 | 0.82 | 0.82 | 0.84 | 0.84 | 0.8034 |
|  | MI | 0.78 | 0.78 | 0.79 | 0.79 | 0.79 | 0.79 | 0.79 | 0.79 | 0.80 | 0.79 | 0.80 | 0.80 | 0.80 | 0.81 | 0.81 | 0.82 | 0.82 | 0.82 | 0.84 | 0.84 | 0.8030 |
| 21 | CIM | 0.86 | 0.87 | 0.87 | 0.87 | 0.87 | 0.87 | 0.87 | 0.87 | 0.87 | 0.87 | 0.87 | 0.87 | 0.88 | 0.88 | 0.88 | 0.88 | 0.89 | 0.90 | 0.89 | 0.90 | 0.8783 |
|  | EM | 0.86 | 0.86 | 0.87 | 0.87 | 0.87 | 0.87 | 0.87 | 0.87 | 0.87 | 0.87 | 0.87 | 0.87 | 0.87 | 0.87 | 0.88 | 0.88 | 0.88 | 0.89 | 0.89 | 0.89 | 0.8740 |
|  | MI | 0.86 | 0.86 | 0.87 | 0.87 | 0.87 | 0.87 | 0.87 | 0.87 | 0.87 | 0.87 | 0.87 | 0.87 | 0.87 | 0.87 | 0.88 | 0.88 | 0.88 | 0.89 | 0.89 | 0.89 | 0.8741 |
| 22 | CIM | 0.81 | 0.81 | 0.81 | 0.82 | 0.81 | 0.82 | 0.82 | 0.82 | 0.82 | 0.82 | 0.82 | 0.82 | 0.82 | 0.83 | 0.83 | 0.84 | 0.85 | 0.85 | 0.85 | 0.86 | 0.8269 |
|  | EM | 0.81 | 0.81 | 0.81 | 0.82 | 0.82 | 0.82 | 0.82 | 0.82 | 0.82 | 0.82 | 0.82 | 0.83 | 0.83 | 0.83 | 0.84 | 0.85 | 0.86 | 0.85 | 0.87 | 0.87 | 0.8318 |
|  | MI | 0.81 | 0.81 | 0.81 | 0.82 | 0.82 | 0.82 | 0.82 | 0.82 | 0.82 | 0.82 | 0.82 | 0.82 | 0.83 | 0.83 | 0.84 | 0.85 | 0.86 | 0.85 | 0.87 | 0.87 | 0.8316 |
| 23 | CIM | 0.59 | 0.59 | 0.60 | 0.60 | 0.61 | 0.61 | 0.61 | 0.61 | 0.62 | 0.62 | 0.62 | 0.63 | 0.63 | 0.64 | 0.65 | 0.67 | 0.69 | 0.71 | 0.72 | 0.73 | 0.6395 |
|  | EM | 0.59 | 0.59 | 0.60 | 0.60 | 0.60 | 0.61 | 0.61 | 0.61 | 0.62 | 0.62 | 0.62 | 0.62 | 0.62 | 0.64 | 0.65 | 0.66 | 0.69 | 0.71 | 0.71 | 0.71 | 0.6357 |
|  | MI | 0.59 | 0.59 | 0.60 | 0.60 | 0.60 | 0.61 | 0.61 | 0.61 | 0.62 | 0.62 | 0.62 | 0.62 | 0.62 | 0.64 | 0.64 | 0.66 | 0.68 | 0.70 | 0.70 | 0.71 | 0.6344 |
| 24 | CIM | 0.83 | 0.83 | 0.83 | 0.83 | 0.83 | 0.84 | 0.83 | 0.84 | 0.84 | 0.84 | 0.83 | 0.84 | 0.84 | 0.84 | 0.84 | 0.85 | 0.85 | 0.85 | 0.83 | 0.83 | 0.8380 |
|  | EM | 0.83 | 0.83 | 0.83 | 0.83 | 0.83 | 0.84 | 0.84 | 0.84 | 0.84 | 0.84 | 0.84 | 0.84 | 0.84 | 0.84 | 0.85 | 0.85 | 0.85 | 0.85 | 0.86 | 0.86 | 0.8426 |
|  | MI | 0.83 | 0.83 | 0.83 | 0.83 | 0.83 | 0.84 | 0.84 | 0.84 | 0.84 | 0.84 | 0.84 | 0.84 | 0.84 | 0.84 | 0.85 | 0.85 | 0.85 | 0.85 | 0.86 | 0.86 | 0.8424 |
| 25 | CIM | 0.86 | 0.86 | 0.86 | 0.86 | 0.86 | 0.86 | 0.85 | 0.86 | 0.86 | 0.86 | 0.85 | 0.86 | 0.85 | 0.85 | 0.85 | 0.85 | 0.86 | 0.85 | 0.85 | 0.85 | 0.8543 |
|  | EM | 0.86 | 0.86 | 0.86 | 0.86 | 0.86 | 0.86 | 0.86 | 0.87 | 0.87 | 0.87 | 0.87 | 0.87 | 0.87 | 0.87 | 0.88 | 0.88 | 0.89 | 0.89 | 0.90 | 0.90 | 0.8735 |
|  | MI | 0.86 | 0.86 | 0.86 | 0.86 | 0.86 | 0.86 | 0.86 | 0.87 | 0.87 | 0.87 | 0.87 | 0.87 | 0.87 | 0.87 | 0.88 | 0.88 | 0.89 | 0.89 | 0.90 | 0.90 | 0.8734 |
| 26 | CIM | 0.86 | 0.86 | 0.86 | 0.86 | 0.86 | 0.86 | 0.86 | 0.86 | 0.85 | 0.86 | 0.85 | 0.85 | 0.85 | 0.85 | 0.85 | 0.85 | 0.85 | 0.85 | 0.84 | 0.85 | 0.8537 |
|  | EM | 0.86 | 0.86 | 0.86 | 0.87 | 0.87 | 0.87 | 0.87 | 0.87 | 0.87 | 0.87 | 0.87 | 0.87 | 0.87 | 0.88 | 0.88 | 0.89 | 0.89 | 0.90 | 0.90 | 0.90 | 0.8757 |
|  | MI | 0.86 | 0.86 | 0.86 | 0.87 | 0.87 | 0.87 | 0.87 | 0.87 | 0.87 | 0.87 | 0.87 | 0.87 | 0.87 | 0.88 | 0.88 | 0.88 | 0.89 | 0.90 | 0.90 | 0.91 | 0.8756 |
| 27 | CIM | 0.76 | 0.77 | 0.77 | 0.78 | 0.78 | 0.78 | 0.78 | 0.78 | 0.78 | 0.79 | 0.78 | 0.79 | 0.79 | 0.80 | 0.81 | 0.82 | 0.83 | 0.84 | 0.85 | 0.85 | 0.7985 |
|  | EM | 0.76 | 0.77 | 0.77 | 0.77 | 0.78 | 0.78 | 0.77 | 0.78 | 0.78 | 0.78 | 0.78 | 0.78 | 0.78 | 0.79 | 0.80 | 0.81 | 0.81 | 0.83 | 0.83 | 0.83 | 0.7908 |
|  | MI | 0.76 | 0.77 | 0.77 | 0.77 | 0.77 | 0.78 | 0.77 | 0.78 | 0.78 | 0.78 | 0.78 | 0.78 | 0.78 | 0.79 | 0.80 | 0.81 | 0.81 | 0.83 | 0.83 | 0.84 | 0.7908 |
| 28 | CIM | 0.87 | 0.87 | 0.87 | 0.86 | 0.87 | 0.86 | 0.87 | 0.86 | 0.86 | 0.86 | 0.86 | 0.86 | 0.86 | 0.86 | 0.86 | 0.87 | 0.87 | 0.87 | 0.85 | 0.86 | 0.8632 |
|  | EM | 0.87 | 0.87 | 0.87 | 0.87 | 0.87 | 0.87 | 0.88 | 0.87 | 0.88 | 0.88 | 0.88 | 0.88 | 0.88 | 0.88 | 0.89 | 0.89 | 0.89 | 0.89 | 0.90 | 0.90 | 0.8812 |
|  | MI | 0.87 | 0.87 | 0.87 | 0.87 | 0.87 | 0.87 | 0.88 | 0.87 | 0.87 | 0.88 | 0.88 | 0.88 | 0.88 | 0.88 | 0.89 | 0.89 | 0.89 | 0.89 | 0.90 | 0.90 | 0.8810 |
| 29 | CIM | 0.86 | 0.86 | 0.86 | 0.86 | 0.86 | 0.86 | 0.86 | 0.86 | 0.86 | 0.86 | 0.86 | 0.86 | 0.86 | 0.87 | 0.87 | 0.87 | 0.87 | 0.88 | 0.88 | 0.88 | 0.8650 |
|  | EM | 0.86 | 0.86 | 0.86 | 0.86 | 0.86 | 0.86 | 0.86 | 0.86 | 0.86 | 0.86 | 0.86 | 0.86 | 0.86 | 0.87 | 0.87 | 0.87 | 0.87 | 0.88 | 0.88 | 0.87 | 0.8645 |
|  | MI | 0.86 | 0.86 | 0.86 | 0.86 | 0.86 | 0.86 | 0.86 | 0.86 | 0.86 | 0.86 | 0.86 | 0.86 | 0.86 | 0.87 | 0.87 | 0.87 | 0.87 | 0.88 | 0.88 | 0.88 | 0.8646 |
| 30 | CIM | 0.63 | 0.64 | 0.64 | 0.64 | 0.65 | 0.65 | 0.66 | 0.66 | 0.66 | 0.66 | 0.66 | 0.67 | 0.68 | 0.69 | 0.70 | 0.72 | 0.73 | 0.75 | 0.69 | 0.71 | 0.6756 |
|  | EM | 0.63 | 0.64 | 0.64 | 0.64 | 0.64 | 0.65 | 0.65 | 0.65 | 0.65 | 0.65 | 0.65 | 0.65 | 0.67 | 0.67 | 0.68 | 0.69 | 0.71 | 0.73 | 0.74 | 0.75 | 0.6706 |
|  | MI | 0.63 | 0.63 | 0.64 | 0.64 | 0.64 | 0.64 | 0.65 | 0.65 | 0.65 | 0.65 | 0.65 | 0.65 | 0.67 | 0.67 | 0.68 | 0.69 | 0.71 | 0.73 | 0.74 | 0.75 | 0.6700 |
| 31 | CIM | 0.48 | 0.49 | 0.49 | 0.50 | 0.51 | 0.51 | 0.51 | 0.51 | 0.52 | 0.52 | 0.53 | 0.53 | 0.54 | 0.55 | 0.57 | 0.58 | 0.61 | 0.64 | 0.66 | 0.66 | 0.5490 |
|  | EM | 0.48 | 0.49 | 0.49 | 0.50 | 0.50 | 0.50 | 0.50 | 0.51 | 0.51 | 0.51 | 0.52 | 0.51 | 0.53 | 0.54 | 0.55 | 0.56 | 0.58 | 0.61 | 0.64 | 0.62 | 0.5363 |
|  | MI | 0.48 | 0.49 | 0.49 | 0.50 | 0.50 | 0.50 | 0.50 | 0.51 | 0.51 | 0.51 | 0.52 | 0.51 | 0.53 | 0.53 | 0.55 | 0.56 | 0.58 | 0.61 | 0.63 | 0.62 | 0.5347 |
| 32 | CIM | 0.85 | 0.85 | 0.85 | 0.85 | 0.85 | 0.85 | 0.84 | 0.85 | 0.85 | 0.85 | 0.85 | 0.85 | 0.85 | 0.85 | 0.86 | 0.86 | 0.86 | 0.86 | 0.85 | 0.87 | 0.8513 |
|  | EM | 0.85 | 0.85 | 0.85 | 0.85 | 0.85 | 0.85 | 0.85 | 0.85 | 0.86 | 0.86 | 0.86 | 0.86 | 0.86 | 0.86 | 0.87 | 0.87 | 0.88 | 0.88 | 0.88 | 0.89 | 0.8622 |
|  | MI | 0.85 | 0.85 | 0.85 | 0.85 | 0.85 | 0.85 | 0.85 | 0.85 | 0.86 | 0.86 | 0.86 | 0.86 | 0.86 | 0.86 | 0.87 | 0.87 | 0.88 | 0.88 | 0.88 | 0.89 | 0.8622 |
| 33 | CIM | 0.84 | 0.84 | 0.84 | 0.84 | 0.84 | 0.84 | 0.84 | 0.85 | 0.85 | 0.85 | 0.85 | 0.85 | 0.85 | 0.85 | 0.85 | 0.86 | 0.86 | 0.87 | 0.87 | 0.88 | 0.8522 |
|  | EM | 0.84 | 0.84 | 0.84 | 0.84 | 0.84 | 0.85 | 0.85 | 0.85 | 0.85 | 0.85 | 0.85 | 0.85 | 0.85 | 0.86 | 0.86 | 0.87 | 0.87 | 0.88 | 0.89 | 0.89 | 0.8577 |
|  | MI | 0.84 | 0.84 | 0.84 | 0.84 | 0.84 | 0.85 | 0.85 | 0.85 | 0.85 | 0.85 | 0.85 | 0.85 | 0.85 | 0.86 | 0.86 | 0.87 | 0.87 | 0.88 | 0.89 | 0.89 | 0.8575 |
| 34 | CIM | 0.87 | 0.87 | 0.87 | 0.87 | 0.87 | 0.87 | 0.87 | 0.87 | 0.87 | 0.88 | 0.87 | 0.87 | 0.88 | 0.88 | 0.88 | 0.88 | 0.89 | 0.89 | 0.90 | 0.89 | 0.8783 |
|  | EM | 0.87 | 0.87 | 0.87 | 0.87 | 0.87 | 0.87 | 0.87 | 0.87 | 0.87 | 0.87 | 0.87 | 0.87 | 0.88 | 0.88 | 0.88 | 0.88 | 0.89 | 0.89 | 0.89 | 0.89 | 0.8772 |
|  | MI | 0.87 | 0.87 | 0.87 | 0.87 | 0.87 | 0.87 | 0.87 | 0.87 | 0.87 | 0.87 | 0.87 | 0.87 | 0.88 | 0.88 | 0.88 | 0.88 | 0.89 | 0.89 | 0.89 | 0.89 | 0.8771 |
| 35 | CIM | 0.85 | 0.85 | 0.85 | 0.85 | 0.85 | 0.85 | 0.85 | 0.85 | 0.85 | 0.85 | 0.84 | 0.85 | 0.85 | 0.84 | 0.85 | 0.85 | 0.85 | 0.85 | 0.83 | 0.83 | 0.8456 |
|  | EM | 0.85 | 0.85 | 0.85 | 0.86 | 0.86 | 0.86 | 0.86 | 0.86 | 0.86 | 0.86 | 0.86 | 0.86 | 0.86 | 0.87 | 0.87 | 0.87 | 0.88 | 0.88 | 0.89 | 0.88 | 0.8657 |
|  | MI | 0.85 | 0.85 | 0.85 | 0.85 | 0.86 | 0.86 | 0.86 | 0.86 | 0.86 | 0.86 | 0.86 | 0.86 | 0.86 | 0.87 | 0.87 | 0.87 | 0.88 | 0.88 | 0.89 | 0.89 | 0.8658 |
| 36 | CIM | 0.73 | 0.73 | 0.73 | 0.74 | 0.74 | 0.74 | 0.74 | 0.74 | 0.74 | 0.75 | 0.75 | 0.75 | 0.75 | 0.76 | 0.77 | 0.78 | 0.79 | 0.80 | 0.80 | 0.81 | 0.7584 |
|  | EM | 0.73 | 0.73 | 0.73 | 0.73 | 0.73 | 0.73 | 0.74 | 0.74 | 0.74 | 0.74 | 0.74 | 0.74 | 0.74 | 0.75 | 0.75 | 0.76 | 0.77 | 0.78 | 0.79 | 0.78 | 0.7491 |
|  | MI | 0.73 | 0.73 | 0.73 | 0.73 | 0.73 | 0.73 | 0.74 | 0.74 | 0.74 | 0.74 | 0.74 | 0.74 | 0.74 | 0.75 | 0.75 | 0.76 | 0.77 | 0.78 | 0.79 | 0.79 | 0.7492 |
| 37 | CIM | 0.66 | 0.66 | 0.67 | 0.67 | 0.67 | 0.67 | 0.68 | 0.68 | 0.68 | 0.68 | 0.68 | 0.68 | 0.69 | 0.70 | 0.70 | 0.71 | 0.73 | 0.73 | 0.74 | 0.75 | 0.6931 |
|  | EM | 0.66 | 0.66 | 0.67 | 0.67 | 0.68 | 0.68 | 0.68 | 0.68 | 0.68 | 0.69 | 0.69 | 0.69 | 0.70 | 0.71 | 0.72 | 0.72 | 0.75 | 0.75 | 0.77 | 0.78 | 0.7044 |
|  | MI | 0.66 | 0.66 | 0.67 | 0.67 | 0.68 | 0.68 | 0.68 | 0.68 | 0.68 | 0.69 | 0.69 | 0.69 | 0.70 | 0.71 | 0.72 | 0.72 | 0.74 | 0.75 | 0.77 | 0.78 | 0.7038 |
| 38 | CIM | 0.87 | 0.87 | 0.87 | 0.87 | 0.87 | 0.87 | 0.87 | 0.87 | 0.88 | 0.88 | 0.88 | 0.88 | 0.88 | 0.88 | 0.88 | 0.89 | 0.89 | 0.89 | 0.89 | 0.90 | 0.8794 |
|  | EM | 0.87 | 0.87 | 0.87 | 0.87 | 0.87 | 0.87 | 0.88 | 0.87 | 0.88 | 0.88 | 0.88 | 0.88 | 0.88 | 0.88 | 0.89 | 0.89 | 0.89 | 0.90 | 0.90 | 0.90 | 0.8826 |
|  | MI | 0.87 | 0.87 | 0.87 | 0.87 | 0.87 | 0.87 | 0.88 | 0.87 | 0.88 | 0.88 | 0.88 | 0.88 | 0.88 | 0.88 | 0.89 | 0.89 | 0.89 | 0.90 | 0.90 | 0.90 | 0.8825 |
| 39 | CIM | 0.76 | 0.77 | 0.77 | 0.77 | 0.77 | 0.78 | 0.78 | 0.78 | 0.78 | 0.78 | 0.78 | 0.78 | 0.79 | 0.79 | 0.81 | 0.81 | 0.82 | 0.83 | 0.83 | 0.84 | 0.7929 |
|  | EM | 0.76 | 0.77 | 0.77 | 0.77 | 0.77 | 0.77 | 0.77 | 0.77 | 0.77 | 0.78 | 0.77 | 0.77 | 0.78 | 0.78 | 0.79 | 0.79 | 0.79 | 0.80 | 0.81 | 0.80 | 0.7800 |
|  | MI | 0.76 | 0.77 | 0.77 | 0.77 | 0.77 | 0.77 | 0.77 | 0.77 | 0.77 | 0.78 | 0.77 | 0.77 | 0.78 | 0.78 | 0.79 | 0.79 | 0.79 | 0.80 | 0.81 | 0.81 | 0.7803 |
| 40 | CIM | 0.81 | 0.81 | 0.81 | 0.82 | 0.82 | 0.82 | 0.82 | 0.82 | 0.82 | 0.82 | 0.82 | 0.82 | 0.83 | 0.83 | 0.84 | 0.84 | 0.85 | 0.86 | 0.87 | 0.87 | 0.8309 |
|  | EM | 0.81 | 0.81 | 0.81 | 0.82 | 0.81 | 0.82 | 0.82 | 0.82 | 0.82 | 0.82 | 0.82 | 0.82 | 0.82 | 0.82 | 0.83 | 0.83 | 0.84 | 0.84 | 0.85 | 0.85 | 0.8246 |
|  | MI | 0.81 | 0.81 | 0.81 | 0.82 | 0.82 | 0.82 | 0.82 | 0.82 | 0.82 | 0.82 | 0.82 | 0.82 | 0.82 | 0.82 | 0.83 | 0.83 | 0.84 | 0.84 | 0.85 | 0.85 | 0.8245 |
| 41 | CIM | 0.92 | 0.92 | 0.92 | 0.91 | 0.91 | 0.91 | 0.90 | 0.90 | 0.90 | 0.90 | 0.90 | 0.90 | 0.89 | 0.88 | 0.87 | 0.86 | 0.85 | 0.85 | 0.78 | 0.79 | 0.8803 |
|  | EM | 0.92 | 0.92 | 0.92 | 0.93 | 0.93 | 0.93 | 0.93 | 0.93 | 0.93 | 0.93 | 0.93 | 0.93 | 0.93 | 0.93 | 0.94 | 0.94 | 0.94 | 0.94 | 0.94 | 0.94 | 0.9316 |
|  | MI | 0.92 | 0.92 | 0.92 | 0.93 | 0.93 | 0.93 | 0.93 | 0.93 | 0.93 | 0.93 | 0.93 | 0.93 | 0.93 | 0.93 | 0.94 | 0.94 | 0.94 | 0.94 | 0.94 | 0.95 | 0.9320 |
| 42 | CIM | 0.85 | 0.85 | 0.85 | 0.84 | 0.85 | 0.85 | 0.85 | 0.85 | 0.85 | 0.84 | 0.85 | 0.85 | 0.85 | 0.85 | 0.85 | 0.85 | 0.85 | 0.85 | 0.84 | 0.84 | 0.8465 |
|  | EM | 0.85 | 0.85 | 0.85 | 0.85 | 0.85 | 0.85 | 0.85 | 0.85 | 0.85 | 0.85 | 0.86 | 0.86 | 0.86 | 0.86 | 0.86 | 0.87 | 0.87 | 0.87 | 0.88 | 0.88 | 0.8590 |
|  | MI | 0.85 | 0.85 | 0.85 | 0.85 | 0.85 | 0.85 | 0.85 | 0.85 | 0.85 | 0.85 | 0.86 | 0.86 | 0.86 | 0.86 | 0.86 | 0.87 | 0.87 | 0.87 | 0.88 | 0.88 | 0.8589 |
| 43 | CIM | 0.80 | 0.80 | 0.80 | 0.80 | 0.80 | 0.80 | 0.80 | 0.80 | 0.80 | 0.80 | 0.80 | 0.80 | 0.80 | 0.80 | 0.80 | 0.80 | 0.80 | 0.81 | 0.80 | 0.81 | 0.8012 |
|  | EM | 0.80 | 0.81 | 0.81 | 0.81 | 0.81 | 0.81 | 0.81 | 0.82 | 0.82 | 0.82 | 0.82 | 0.82 | 0.82 | 0.83 | 0.84 | 0.84 | 0.85 | 0.86 | 0.86 | 0.88 | 0.8288 |
|  | MI | 0.80 | 0.81 | 0.81 | 0.81 | 0.81 | 0.81 | 0.81 | 0.82 | 0.82 | 0.82 | 0.82 | 0.82 | 0.82 | 0.83 | 0.84 | 0.84 | 0.85 | 0.86 | 0.86 | 0.88 | 0.8286 |
| 44 | CIM | 0.85 | 0.84 | 0.84 | 0.84 | 0.84 | 0.84 | 0.83 | 0.83 | 0.83 | 0.83 | 0.83 | 0.82 | 0.83 | 0.83 | 0.81 | 0.81 | 0.81 | 0.80 | 0.77 | 0.76 | 0.8206 |
|  | EM | 0.85 | 0.85 | 0.85 | 0.85 | 0.85 | 0.85 | 0.85 | 0.86 | 0.86 | 0.86 | 0.86 | 0.86 | 0.86 | 0.87 | 0.87 | 0.87 | 0.88 | 0.88 | 0.88 | 0.88 | 0.8621 |
|  | MI | 0.85 | 0.85 | 0.85 | 0.85 | 0.85 | 0.85 | 0.85 | 0.86 | 0.86 | 0.86 | 0.86 | 0.86 | 0.86 | 0.86 | 0.87 | 0.87 | 0.88 | 0.88 | 0.88 | 0.89 | 0.8627 |
| 45 | CIM | 0.80 | 0.80 | 0.81 | 0.81 | 0.81 | 0.81 | 0.81 | 0.81 | 0.82 | 0.82 | 0.82 | 0.82 | 0.82 | 0.83 | 0.83 | 0.84 | 0.85 | 0.86 | 0.86 | 0.87 | 0.8264 |
|  | EM | 0.80 | 0.80 | 0.80 | 0.80 | 0.81 | 0.80 | 0.80 | 0.80 | 0.81 | 0.81 | 0.81 | 0.81 | 0.81 | 0.81 | 0.81 | 0.82 | 0.82 | 0.82 | 0.82 | 0.82 | 0.8095 |
|  | MI | 0.80 | 0.80 | 0.80 | 0.80 | 0.81 | 0.80 | 0.80 | 0.80 | 0.80 | 0.81 | 0.81 | 0.81 | 0.81 | 0.81 | 0.81 | 0.82 | 0.82 | 0.82 | 0.82 | 0.83 | 0.8099 |
| 46 | CIM | 0.81 | 0.81 | 0.81 | 0.81 | 0.81 | 0.81 | 0.81 | 0.81 | 0.82 | 0.81 | 0.81 | 0.81 | 0.81 | 0.81 | 0.82 | 0.81 | 0.81 | 0.82 | 0.83 | 0.83 | 0.8137 |
|  | EM | 0.81 | 0.81 | 0.81 | 0.81 | 0.82 | 0.82 | 0.82 | 0.82 | 0.82 | 0.82 | 0.83 | 0.82 | 0.83 | 0.83 | 0.84 | 0.84 | 0.85 | 0.85 | 0.86 | 0.87 | 0.8307 |
|  | MI | 0.81 | 0.81 | 0.81 | 0.81 | 0.82 | 0.82 | 0.82 | 0.82 | 0.83 | 0.82 | 0.83 | 0.82 | 0.83 | 0.83 | 0.84 | 0.84 | 0.85 | 0.85 | 0.86 | 0.87 | 0.8306 |
| 47 | CIM | 0.34 | 0.35 | 0.35 | 0.36 | 0.36 | 0.37 | 0.37 | 0.38 | 0.38 | 0.39 | 0.39 | 0.39 | 0.39 | 0.42 | 0.44 | 0.47 | 0.49 | 0.53 | 0.54 | 0.56 | 0.4173 |
|  | EM | 0.34 | 0.34 | 0.35 | 0.35 | 0.35 | 0.36 | 0.36 | 0.36 | 0.36 | 0.37 | 0.36 | 0.37 | 0.36 | 0.39 | 0.39 | 0.41 | 0.43 | 0.46 | 0.47 | 0.46 | 0.3852 |
|  | MI | 0.34 | 0.34 | 0.35 | 0.35 | 0.35 | 0.36 | 0.36 | 0.36 | 0.36 | 0.37 | 0.36 | 0.37 | 0.37 | 0.39 | 0.40 | 0.42 | 0.43 | 0.46 | 0.47 | 0.46 | 0.3856 |
| 48 | CIM | 0.78 | 0.78 | 0.78 | 0.78 | 0.79 | 0.79 | 0.79 | 0.79 | 0.79 | 0.79 | 0.79 | 0.79 | 0.80 | 0.80 | 0.81 | 0.82 | 0.83 | 0.84 | 0.75 | 0.76 | 0.7942 |
|  | EM | 0.78 | 0.78 | 0.78 | 0.78 | 0.79 | 0.79 | 0.79 | 0.79 | 0.79 | 0.79 | 0.79 | 0.79 | 0.80 | 0.80 | 0.81 | 0.82 | 0.82 | 0.84 | 0.84 | 0.84 | 0.8026 |
|  | MI | 0.78 | 0.78 | 0.78 | 0.78 | 0.79 | 0.79 | 0.79 | 0.79 | 0.79 | 0.79 | 0.79 | 0.79 | 0.80 | 0.80 | 0.81 | 0.82 | 0.82 | 0.84 | 0.84 | 0.84 | 0.8024 |
| 49 | CIM | 0.72 | 0.72 | 0.73 | 0.73 | 0.73 | 0.73 | 0.73 | 0.74 | 0.74 | 0.74 | 0.75 | 0.75 | 0.75 | 0.76 | 0.77 | 0.78 | 0.80 | 0.80 | 0.78 | 0.81 | 0.7539 |
|  | EM | 0.72 | 0.72 | 0.72 | 0.72 | 0.73 | 0.72 | 0.72 | 0.73 | 0.73 | 0.73 | 0.74 | 0.74 | 0.74 | 0.75 | 0.76 | 0.77 | 0.77 | 0.77 | 0.78 | 0.80 | 0.7458 |
|  | MI | 0.72 | 0.72 | 0.72 | 0.72 | 0.73 | 0.72 | 0.72 | 0.73 | 0.73 | 0.73 | 0.74 | 0.74 | 0.74 | 0.75 | 0.76 | 0.76 | 0.77 | 0.77 | 0.78 | 0.80 | 0.7456 |
| 50 | CIM | 0.76 | 0.76 | 0.76 | 0.76 | 0.76 | 0.76 | 0.76 | 0.76 | 0.76 | 0.76 | 0.76 | 0.76 | 0.75 | 0.75 | 0.75 | 0.76 | 0.76 | 0.76 | 0.72 | 0.74 | 0.7560 |
|  | EM | 0.76 | 0.77 | 0.77 | 0.77 | 0.77 | 0.77 | 0.78 | 0.78 | 0.78 | 0.78 | 0.79 | 0.79 | 0.79 | 0.80 | 0.80 | 0.81 | 0.83 | 0.84 | 0.84 | 0.84 | 0.7950 |
|  | MI | 0.76 | 0.77 | 0.77 | 0.77 | 0.77 | 0.77 | 0.78 | 0.78 | 0.78 | 0.78 | 0.79 | 0.79 | 0.79 | 0.80 | 0.80 | 0.81 | 0.83 | 0.83 | 0.84 | 0.85 | 0.7951 |
| **WHOLE** | CIM | 0.77 | 0.78 | 0.78 | 0.78 | 0.78 | 0.78 | 0.78 | 0.78 | 0.78 | 0.78 | 0.78 | 0.79 | 0.79 | 0.79 | 0.80 | 0.80 | 0.81 | 0.82 | 0.80 | 0.81 | **0.78955** |
|  | EM | 0.77 | 0.78 | 0.78 | 0.78 | 0.78 | 0.78 | 0.78 | 0.79 | 0.79 | 0.79 | 0.79 | 0.79 | 0.79 | 0.80 | 0.80 | 0.81 | 0.82 | 0.83 | 0.83 | 0.83 | **0.79646** |
|  | MI | 0.77 | 0.78 | 0.78 | 0.78 | 0.78 | 0.78 | 0.78 | 0.79 | 0.79 | 0.79 | 0.79 | 0.79 | 0.79 | 0.80 | 0.80 | 0.81 | 0.82 | 0.82 | 0.83 | 0.84 | **0.79630** |
