# Supplementary material for: The impact of missing data rates and imputation methods on the assumption of unidimensionality
Source: PLoS One. 2025 Apr 30;20(4):e0321344. doi: 10.1371/journal.pone.0321344 (PMC12043241; doi:10.1371/journal.pone.0321344)
Supplement: Attachment — Submitted filename: PONE-D-24-57472.pdf [file pone.0321344.s004.pdf]

The Impact of Missing Data Rates and Imputation Methods on the Assumption of Unidimensionality

The paper examined the impact of missing data rates and imputation methods on fulfilling the assumption of unidimensionality, a core assumption supporting many statistical models. The importance/significance of conducting such study is not clearly highlighted in the paper. There are several similar studies that have been conducted, for instance the reference [19] has compared 6 methods for handling missing data, [49] compared 12 imputation techniques, and [30] in which 4 imputation techniques have been compared; comparing and highlighting their differences in a section (related work) would perhaps justify the motivation of conducting the study.

A clear formal definition of unidimensionality should be given. A section on motivation of the study should be included to justify the significance of conducting the analysis.

In the paper, the authors have examined three methods for imputing missing values, namely: *multiple imputation* (MI), *expectation maximization algorithm* (EM), and *corrected item mean* (CIM). Authors should deliberate on all possible methods for imputing missing values and justify the reasons for focusing only on the above three methods. Also, there are three mechanisms of missing data as defined in [17] that are *Missing Completely At Random* (MCAR), *Missing At Random* (MAR), and *Missing Not at Random*. While the study has assumed MCAR with missing rates of 1% … 50%. Justification on the selection of mechanism, i.e. MCAR, and the range of missing values is not clear. Why 50% is set as the highest missing rate? Also, the selection of 5000 examinees and a test of 50 items is not obvious. Moreover, performing the analysis on real dataset might result in more profound results.

The discussion section is not well written as it merely reports the findings with regard to *Cronbach's* α, *CITC*, *Eigenvalues*, *CTV*, and *communalities* which have been reported in the earlier sections. Moreover, the findings and discussions are based on the selected imputation techniques. It is not comprehensive as there might be other imputation techniques that would show better results.

There are several typo/grammar mistakes as follows:

(a) Inconsistent spelling of terms, e.g. unidimensionality; communalities; components;

(b) Different style of citation is used in the following sentence, "… which weakens the predictive power (Smith, 2002)."

(c) " … Eigenvalues or Residuals, He also mentioned a group of Indices Based on Factor Analysis, …"; improper punctuation mark before He.

(d) Check the use of lowercase and uppercase letters. Some samples are as follows: "… univariate Pattern when missing occurs with only one variable, multivariate pattern When there is more than one variable containing missing data [11], …"; "Rubin [17] proposed three mechanisms of missing data, The first mechanism is Missing Completely At Random (MCAR), …"; etc.

(e) Variables written in text should use the same notation as used in the equation. See for instance Pmi in text and PM_i in equation. Imj in text and IM_j in equation.

(f) to explores → explore

(g) " … from (0.99169) at (2%) missing rate to (0.994831) at (50%) …" → 0.991690
